# A Single-Cell Omics Network Model of Cell Crosstalk during the Formation of Primordial Follicles

**DOI:** 10.3390/cells11030332

**Published:** 2022-01-20

**Authors:** Qian Wang, Ang Dong, Libo Jiang, Christopher Griffin, Rongling Wu

**Affiliations:** 1 Department of Obstetrics and Gynecology, Tianjin Medical University General Hospital, Tianjin 300052, China; 2 Tianjin Key Laboratory of Female Reproductive Health and Eugenics, Tianjin 300052, China; 3 Center for Computational Biology, College of Biological Sciences and Technology, Beijing Forestry University, Beijing 100083, China; fantasys05227@163.com (A.D.); jianglibo0534@163.com (L.J.); 4 Applied Research Laboratory, The Pennsylvania State University, University Park, PA 16802, USA; cxg286@psu.edu; 5 Center for Statistical Genetics, Departments of Public Health Sciences and Statistics, The Pennsylvania State University, Hershey, PA 17033, USA

**Keywords:** primordial follicle, evolutionary game theory, fetal germ cell-soma interaction, gene regulatory network modeling, niche index

## Abstract

The fate of fetal germ cells (FGCs) in primordial follicles is largely determined by how they interact with the surrounding granulosa cells. However, the molecular mechanisms underlying this interactive process remain poorly understood. Here, we develop a computational model to characterize how individual genes program and rewire cellular crosstalk across FGCs and somas, how gene regulatory networks mediate signaling pathways that functionally link these two cell types, and how different FGCs diversify and evolve through cooperation and competition during embryo development. We analyze single-cell RNA-seq data of human female embryos using the new model, identifying previously uncharacterized mechanisms behind follicle development. The majority of genes (70%) promote FGC–soma synergism, only with a small portion (4%) that incur antagonism; hub genes function reciprocally between the FGC network and soma network; and germ cells tend to cooperate between different stages of development but compete in the same stage within a developmental embryo. Our network model could serve as a powerful tool to unravel the genomic signatures that mediate folliculogenesis from single-cell omics data.

## 1. Introduction

Primordial follicles of a female embryo, as women’s life-long fertility reserve, have been a longstanding focus of research in reproductive medicine [1,2,3]. The pattern of how primordial follicles develop and function is thought to be species-specific, providing a clue for studying the origin of life [4,5]. A primordial follicle consists of germ cells, surrounded by a single layer of flattened pregranulosa that supports germ cell growth. According to standard reproductive terminologies, germ cells from human female embryos before and after 11 weeks post-fertilization are called primordial germ cells and oogonia, respectively. Following Li et al. [3], we call all of these germ cells fetal germ cells (FGCs) for an easy description. Similarly, gonadal cells that surround FGCs are collectively referred to as somas. Mounting evidence shows that proper FGC development, a key step for organisms to transmit genetic information to the next generation, critically relies on their coordinated interactions with somas [6,7,8,9,10]. The past decade has witnessed the tremendous power of single-cell transcriptional and epigenetic data to monitor the developmental trajectories of FGCs and somas at unprecedented resolution [3,10,11]. Despite the accumulation of single-cell data at an astonishing pace, there is little methodology that can extract and excavate new biological rules underlying FGC–soma interactions from these data. One major reason is that it is difficult to map those complex omics data onto the spatiotemporal heterogeneity of follicle development.

We overcome this challenge by integrating elements of multiple disciplines from ecology, evolution, and game theory. Being a functional complex composed of FGCs and somas [3], a primordial follicle can be viewed as an ecosystem in which these two compartments interact with each other following the principles of community ecology. Allometric scaling of each cell type with the follicle represents a part–whole relationship that can be described by a power equation according to the metabolic theory of ecology [12,13]. We hypothesize that the pattern of FGC–soma interactions can be interpreted through the lens of game theory [14]; i.e., each cell type optimizes its gene expression based on its own strategy and the strategy of its counterpart. One important concept of game theory is the Nash equilibrium [15] where a rational player receives no incremental payoff from changing actions, assuming that other players remain constant in their strategies. To modify the rationality assumption of the Nash equilibrium, Smith and Price [16] proposed the concept of evolutionarily stable strategy. This concept provides a static tool for studying strategy stability in a population in which the frequency of strategies does not change [17].

To characterize how the strategy a player chooses evolves in time, evolutionary game theory uses its own dynamic representation, in which there is no need to define a notion of evolutionary stability; instead, all of the standard stability concepts from dynamic modeling are used [18]. Many explicit time-based models for evolutionary game theory have been developed [17,19,20], some of which have found their remarkable applications to the quantitative genetics of complex traits [21], quantitative epigenetics of maternal-to-zygotic transition [22], and community ecology of interspecific interactions [23]. However, the application of dynamic evolutionary game theory relies on temporal omics data which are extremely difficult to collect for primordial follicles. Single-cell analysis also shows that the transcriptome of FGCs is relatively stable during 4 to 11 weeks post-fertilization due to global epigenomic reprogramming [24], thus providing limited information for dynamic modeling. For these reasons, there is a pressing need to convert steady-state data into an alternate dynamic space for evolutionary game theory to be fully beneficially used.

In this article, we integrate allometric scaling theory and evolutionary game theory into a system of quasi-dynamic ordinary differential equations (qdODEs) for modeling the dynamic change of evolutionarily stable strategies [25]. These qdODEs, whose derivatives are not time-based, capitalize on the pervasive existence of cell heterogeneity within and between embryos [3,24] and can quantitatively model follicle- and embryo-dependent changes in game strategies by FGCs and somas, without need of temporal data. The mathematical and statistical solution of the qdODEs provides a means of investigating how cells communicate through genes across FGCs and somas. The role of genes in regulating cell–cell crosstalk has been increasingly identified by using single-cell RNA sequencing (scRNA-seq) data. For example, Kumar et al. [26] identified important scRNA-seq genes that affect cell–cell communication associated with tumor characteristics. We apply the qdODE game model to analyze the scRNA-seq expression data of a thousand single cells from different female embryos that develop in different phases [3]. Interestingly, our gene-driven game model characterizes the previously unknown mechanisms that guide how cells communicate across FGCs and somas during the normal development of primordial follicles.

## 2. Methods

### 2.1. Niche Index

Suppose we sample a set of *n* human female embryos that develop at the primordial follicle-formation stage of post-fertilization. We monitor the transcriptional profiles of *m* genes that are each expressed in multiple subpopulations of FGCs and multiple subpopulations of somas from the same embryo. The expression level of a specific gene in a cell type is described by the mean of its expression levels over all subpopulations from this cell type. We use $g_{ikj}$ to denote the expression level of gene *j* in a cell type *k* (*k* = 1 for FGC, 2 for somas) from embryo *i*. We view a developing embryo as an ecosystem in which FGCs and somas interact with each other just as two species interact in a shared environment. We define the total of conditions that allow the expression of a gene in an embryo as the niche of the gene. The total expression amount of gene *j* in embryo *i*, calculated as $N_{ij}=\overset{2}{\underset{k=1}{\sum }}g_{ikj}$, and defined as the niche index (NI), reflecting the capacity of this embryo to maintain various resources essential for gene expression. This capacity is similar to that of an ecological habitat that provides a mix of environmental factors for a given species’ survival and growth. According to the magnitude of NI, we serialize *n* embryos in an ascending order and express $g_{ikj}$ as a function of $N_{ij}$, i.e., $g_{kj}\left(N_{ij}\right)$.

### 2.2. Integration of Allometric Scaling Theory and Evolutionary Game Theory

As defined above, $g_{kj}\left(N_{ij}\right)$ describes the individual expression of FGCs or somas, whereas $N_{ij}$ describes the summed expression of these two cell types, which, thus, establish an allometric part–whole relationship. Allometric scaling of the part with the whole can be described by the power equation, expressed as

(1) $$g_{kj}\left(N_{ij}\right)=\alpha_{kj}N_{ij}^{\beta_{kj}}$$

where $\alpha_{kj}$ and $\beta_{kj}$ are the proportionality coefficient and allometric exponent of the power equation for gene *j* expressed in cell type *k*. It is expected that gene expression in different cell types follows distinct power forms determined by these two parameters. Such a distinction is a function of NI, which is used to infer the pattern of interaction between fetal germlines and somas. For example, if $g_{2j}\left(N_{ij}\right)$ > $g_{2j}\left(N_{\left(i+1\right)j}\right)$ in somas is causally related with $g_{1j}\left(N_{ij}\right)$ > $g_{1j}\left(N_{\left(i+1\right)j}\right)$ in FGCs, then we can infer that somatic cells promote FGCs through gene *j*. In contrast, if $g_{2j}\left(N_{ij}\right)$ > $g_{2j}\left(N_{\left(i+1\right)j}\right)$ in somas is causally correlated with $g_{1j}\left(N_{ij}\right)$ < $g_{1j}\left(N_{\left(i+1\right)j}\right)$ in FGCs then somatic cells inhibits fetal germlines through gene *j*. These causal relationships can be characterized by modeling the allometric change of $g_{kj}\left(N_{ij}\right)$ with $N_{ij}$ across embryos through an evolutionary game model.

If FGCs and somas derived from the same embryo are viewed as two interactive players of a game, we use evolutionary game theory to model the strategies by which they interact with each other. Assuming a game with a cooperative/competitive framework (e.g., Hawk-Dove or Prisoner’s Dilemma), game theoretic reasoning suggests that any one player chooses to cooperate or compete with the other player based on their own strategy and the strategy of their interacting partner [14,15]. Taking advantage of the allometric relationship of Equation (1), we extend Smith and Price’s [16] static evolutionary game theory to its (quasi) dynamic representation in order to model how strategies change in populations as a function of the niche index. Dynamic evolutionary game theory can be mathematically formulated as

(2) $$g^{\prime }\left(N_{ij}\right)=\left[\begin{array}{c} \frac{dg_{1j}\left(N_{ij}\right)}{dN_{ij}}\\ \frac{dg_{2j}\left(N_{ij}\right)}{dN_{ij}} \end{array}\right]=\left[\begin{array}{c} Q_{1j}\left(g_{1j}\left(N_{ij}\right):\theta_{1j}\right)+Q_{1\leftarrow 2j}\left(g_{2j}\left(N_{ij}\right):\theta_{1\leftarrow 2j}\right)\\ Q_{2j}\left(g_{2j}\left(N_{ij}\right):\theta_{2j}\right)+Q_{2\leftarrow 1j}\left(g_{1j}\left(N_{ij}\right):\theta_{2\leftarrow 1j}\right) \end{array}\right]$$

where the derivative of $g_{kj}\left(N_{ij}\right)$, the expression level of gene *j* on cell type *k* from embryo *i* (*i* = 1, …, *n*), is partitioned into two components, $Q_{kj}\left(g_{kj}\left(N_{ij}\right):\theta_{kj}\right)$ and $Q_{k\leftarrow k^{\prime }j}\left(g_{k^{\prime }j}\left(N_{ij}\right):\theta_{k\leftarrow k^{\prime }j}\right)$ ($k^{\prime }$ $\neq k,\;k^{\prime }\;$= 1, 2). The first is called the *independent* component that occurs when cell type *k* is assumed to be independent from cell type $k^{\prime }$, whereas the second is the *dependent* component that results from the directional interaction of cell type $k^{\prime }$ with *k*. From a game theoretic perspective, the relative amount of the independent component of a cell type reflects the “payoff” of its self-induced strategy. Relative to the independent component, the dependent component is the “payoff” of a cell type through its reaction to the other cell type. The NI-varying patterns of independent and dependent components are determined by parameters $\theta_{kj}$ and $\theta_{k\leftarrow k^{\prime }j}$, respectively. Unlike classic ODEs with respect to time, ODEs in Equation (2) are specified by the NI derivative, which are thus called quasi-dynamic ODEs or qdODEs [25].

### 2.3. Qualitative and Quantitative Classification of FGC–Soma Interactions

In the Section 2, we describe the statistical procedure for solving qdODEs to obtain the maximum likelihood estimates of $\theta_{kj}$ and $\theta_{k\leftarrow k^{\prime }j}$. Based on these estimates, we can calculate the (first) integrals of $Q_{kj}\left(g_{kj}\left(N_{ij}\right):\theta_{kj}\right)$ and $Q_{k\leftarrow k^{\prime }j}\left(g_{k^{\prime }j}\left(N_{ij}\right):\theta_{k\leftarrow k^{\prime }j}\right)$, denoted as $P_{kj}\left(N_{ij}\right)$ and $P_{k\leftarrow k^{\prime }j}$($N_{ij})$, respectively. These two integral values provide a way to assess the magnitude of gene expression on FGCs or somas and the pattern of how these two cell types interact with each other during embryo development. If $P_{k\leftarrow k^{\prime }j}\left(N_{ij}\right)$ is positive or negative, then this suggests that cell type *k′* activates or inhibits cell type *k*, respectively. If it is zero, this indicates that cell type *k′* is neutral to cell type *k*. Thus, by estimating $P_{k\leftarrow k^{\prime }j}$($N_{ij}$) and $P_{k^{\prime }\leftarrow kj}$($N_{ij}$), we can illustrate the interactive relationship of the two cell types, and more precisely define each pattern of cell–cell interactions (Table 1). If the two cell types activate each other by producing factors that will promote both interacting parties, then synergism occurs. Their relationship would be antagonistic if the two cell types inhibit each other. Directional synergism results if one cell type activates its partner whereas the latter does not affect the former (neutral), while directional antagonism occurs if one cell type inhibits the other and the other is neutral. If one cell type activates the other but the latter inhibits the former, then altruism or exploitation forms. The two cell types may peacefully coexist when they do not regulate each other.

Table 1 summarizes the important features of our computational model in cell–cell interaction detection. The implementation of qdODEs can quantify the strength of each pattern of interaction. The estimation of these interactions is obtained under the maximum likelihood setting. Thus, the maximum payoff to the embryo as a whole can be achieved through the various strategies each cell type uses to interact with the others. Taken together, the definition and interpretation of various patterns of cell–cell interactions can facilitate the exploration of the mass, energetic, or signal basis for each interaction. In addition, as a function of NI, $P_{k\leftarrow k^{\prime }j}$($N_{ij}$) gives the embryo-specific characterization of the FGC–soma interaction and, therefore, establishes an individualized characterization of cell rewiring and communication during biological processes.

### 2.4. Inferring Context-Specific Gene Regulatory Networks from Static Expression Data

We view an FGC or soma as a sample and define the total expression amount of all genes on a sample as the expression index (EI) of the sample. Let *E_ik_* denote the EI of sample *i* from cell type *k* and $g_{j}\left(E_{ik}\right)$ denote the expression level of gene *j* on sample *i* from cell type *k*. We extend the dynamic evolutionary game theory of Equation (2) to include an *m*-dimensional system of qdODEs, which is expressed as

(3) $$g^{\prime }\left(E_{ik}\right)=\left[\begin{array}{c} \frac{dg_{1}\left(E_{ik}\right)}{dE_{ik}}\\ \vdots \\ \frac{dg_{m}\left(E_{ik}\right)}{dE_{ik}} \end{array}\right]=\left[\begin{array}{c} W_{1}\left(g_{1}\left(E_{ik}\right):\psi_{1}\right)+\overset{m}{\underset{j=2}{\sum }}W_{1\leftarrow j}\left(g_{j}\left(E_{ik}\right):\psi_{1\leftarrow j}\right)\;\\ \vdots \\ W_{m}\left(g_{m}\left(E_{ik}\right):\psi_{m}\right)+\overset{m-1}{\underset{j=1}{\sum }}W_{m\leftarrow j}\left(g_{j}\left(E_{ik}\right):\psi_{m\leftarrow j}\right) \end{array}\right]\;$$

where the overall expression level of gene *j* on sample *i* from cell type *k* is specified by its independent expression component $W_{j}\left(\times \right)$ (determined by the strategic capacity of the gene) and aggregate-dependent expression component $\sum W_{j\leftarrow j^{\prime }}\left(\times \right)$ (determined by the interactive strategies implemented by other genes) (*j* = 1, …, *m*; *j*′ = 1, …, *j* − 1, *j* + 1, …, *m*), and $\psi_{j}$ and $\psi_{j\leftarrow j^{\prime }}$ are the ODE parameters that model these components, respectively. 

Equation (3) presents a general framework for capturing gene–gene interactions within a network. Since it is rarely possible that each gene interacts with every other gene in a cell, we need to choose a subset of the most significant genes that interact with each gene. We implement two approaches to infer a sparse gene network. Since the expression level of individual genes is an exponential function of EI (assuming allometric scaling), power equation-based functional clustering [27] is used to classify all genes into different modules based on their functional similarity. Functional modularity theory, widely supported by biological studies [28], is consistent with network community theory [29] in which nodes (i.e., genes) are linked more tightly within than between modules. By regressing the expression value of a focal gene on those of all other genes, we implement LASSO-based variable selection to choose the most significant genes that interact with the focal gene. Through variable selection, the number of genes that are involved in interacting with a focal gene *j* reduces to *d_j_* ($d_{j}\ll m)$, which is gene-dependent. The statistical procedure for solving the reduced qdODEs is given in the Section 2.

### 2.5. Cell–Cell Rewiring by Genes

A single female embryo contains thousands of FGCs in its early stage, only a very small portion of which will successfully develop into oocytes. This process involves the natural selection of FGCs under their mutual cooperation and competition. The detailed network of cell–cell interactions can help to understand the general principle that drives natural selection. Consider *n* FGCs from the same embryo in each of which *m* genes are monitored. Let $g_{cj}$ denote the expression level of gene *j* in FGC *c* (*c* = 1, …, *n*) from an embryo, and define $E_{j}=\overset{n}{\underset{c=1}{\sum }}g_{cj}$ as the EI of the gene over all FGCs. Thus, as a function of $E_{j}$, we express $g_{cj}$ by $g_{c}(E_{j})$. A system of qdODEs that characterize the network of cell–cell interactions is formulated as

(4) $$g^{\prime }\left(E_{j}\right)=\left[\begin{array}{c} \frac{dg_{1}\left(E_{j}\right)}{dE_{j}}\\ \vdots \\ \frac{dg_{n}\left(E_{j}\right)}{dE_{j}} \end{array}\right]=\left[\begin{array}{c} R_{1}\left(g_{1}\left(E_{j}\right):\phi_{1}\right)+\overset{n}{\underset{c=2}{\sum }}R_{1\leftarrow j}\left(g_{c}\left(E_{j}\right):\phi_{1\leftarrow c}\right)\\ \vdots \\ R_{n}\left(g_{n}\left(E_{j}\right):\phi_{n}\right)+\overset{n-1}{\underset{c=1}{\sum }}R_{n\leftarrow c}\left(g_{c}\left(E_{j}\right):\phi_{n\leftarrow c}\right)\; \end{array}\right]\;$$

where the overall gene-specific expression level of FGC *c* is specified by its independent expression component $R_{c}\left(\times \right)$ (assuming that this FGC is in isolation) and aggregate-dependent expression component $\sum R_{c\leftarrow c^{\prime }}\left(\times \right)$ (determined by interactions with other cells) (*c* = 1, …, *n*; *c*′ = 1, …, *c* − 1, *c* + 1, …, *n*), and $\phi_{c}$ and $\phi_{c\leftarrow c^{\prime }}$ are the ODE parameters that model these components, respectively. The statistical procedure described in the Section 2 is modified to solve the qdODEs of Equation (4). By estimating integrals of $R_{c}\left(\times \right)$ and $\sum R_{c\leftarrow c^{\prime }}\left(\times \right)$, we will draw an *n*-node sparse cell–cell network and identify the emergent properties of the network.

### 2.6. Regression Model

Let $y_{j}\left(N_{ij}\right)$ = ${\left(y_{1j}\left(N_{ij}\right),\;y_{2j}\left(N_{ij}\right)\right)}^{T}$ denote the observed expression level of gene *j* in FGCs (1) and somas (2) of embryo *i* (*i* = 1, …, *n*), where $N_{ij}$ is the NI of embryo *i* for gene *j*, defined as the sum of gene expression over the two cell types from the embryo; i.e., $N_{ij}=y_{1j}\left(N_{ij}\right)+y_{2j}\left(N_{ij}\right)$. A regression model of one cell type on the other driven by gene *j*, implemented with the qdODEs from Equation (2), can be formulated as

(5) $$y_{j}\left(N_{ij}\right)=P_{j}\left(N_{ij}\right)+P_{\leftarrow j}\left(N_{ij}\right)+e_{j}\left(N_{ij}\right),\;i=1,\ldots ,\;n\;,$$

where $P_{j}\left(N_{ij}\right)$ = ${\left(P_{1j}\left(N_{ij}\right),\;P_{2j}\left(N_{ij}\right)\right)}^{T}$ is the independent expression component vector of two cell types as a function of $N_{ij}$ for gene j, $P_{\leftarrow j}\left(N_{ij}\right)$ = ${\left(P_{1\leftarrow 2j}\left(N_{ij}\right),\;P_{2\leftarrow 1j}\left(N_{ij}\right)\right)}^{T}$ is the dependent expression component vector of one cell type affected by the second cell type as a function of $N_{ij}$ for gene *j*, and $e_{j}\left(N_{ij}\right)$ = ${\left(e_{1j}\left(N_{ij}\right),\;e_{2j}\left(N_{ij}\right)\right)}^{T}$ is the residual expression error for gene *j*, obeying a bivariate n-dimensional normal distribution with mean vector **0** and sample-dependent covariance matrix $\sum_{j}$ for gene *j*, expressed as

(6) $$\sum_{j}=\left(\begin{array}{cc} \sum_{1j} & \sum_{21j}\\ \sum_{12j} & \sum_{2j} \end{array}\right),\;$$

consisting of $\sum_{1j}$ and $\sum_{2j}$, the NI-varying “longitudinal” residual covariance matrices of the FGCs and somas, respectively, and $\sum_{12j}=\sum_{21j}$, the NI-varying “longitudinal” residual covariance matrix between FGCs and somas.

### 2.7. Likelihood and Test

Let $y_{j}=(y_{j}\left(N_{1j}\right)$, …, $y_{j}\left(N_{nj}\right))$. The likelihood of expression data on two cell types across *n* embryos is formulated as

(7) $$L\left(y_{j}\right)=f_{j}((y_{j}\left(N_{1j}\right),\;\ldots ,\;y_{j}\left(N_{nj}\right)):\mu_{j},\;\sum_{j})\;$$

where $f_{j}\left(\cdot \right)$ is the multivariate normal probability density function with mean vector:

(8) $$\begin{array}{ll} \;\mu_{j} & =(\mu_{j}\left(N_{1j}\right),\;\ldots ,\;\mu_{j}\left(N_{nj}\right))\\ & =((\mu_{1j}\left(N\right),\mu_{2j}\left(N_{1j}\right)),\;\ldots ,\;(\mu_{1j}\left(N_{nj}\right),\mu_{2j}\left(N_{nj}\right)))\\ & =((P_{1j}\left(N_{1j}\right)+P_{1\leftarrow 2j}\left(N_{1j}\right),\;P_{2j}\left(N_{1j}\right)+P_{2\leftarrow 1j}\left(N_{1j}\right)),\;\ldots ,\\ & (P_{1j}\left(N_{nj}\right)+P_{1\leftarrow 2j}\left(N_{nj}\right),\;P_{2j}\left(N_{nj}\right)+P_{2\leftarrow 1j}\left(N_{nj}\right)))\;. \end{array}$$

We implement qdODEs from Equation (2) to model the NI-varying means of gene expression on two cell types in Equation (8) and a bi-variate AR (1) model to fit the structure of matrix $\sum_{j}$. We use a power equation to model the independent expression component and we use a Legendre orthogonal polynomials (LOP)-based nonparametric approach to model the dependent expression component. The fourth-order Runge–Kutta algorithm is used to estimate ODE parameters by maximizing the likelihood (7). 

We develop a statistical procedure for testing whether FGC–soma interaction is significant, driven by gene *j*. Under the assumption of no interaction, we formulate a similar likelihood for Equation (7), but under which the NI-varying means of gene expression are modelled as

(9) $$\begin{array}{ll} \;\mu_{j} & =(\mu_{j}\left(N_{1j}\right),\;\ldots ,\;\mu_{j}\left(N_{nj}\right))\\ & =((\mu_{1j}\left(N_{1j}\right),\mu_{2j}\left(N_{1j}\right)),\;\ldots ,\;(\mu_{1j}\left(N_{nj}\right),\mu_{2j}\left(N_{nj}\right)))\\ & =((P_{1j}\left(N_{1j}\right),\;P_{2j}\left(N_{1j}\right)),\;\ldots ,\;(P_{1j}\left(N_{nj}\right),\;P_{2j}\left(N_{nj}\right)))\; \end{array}$$

where no interaction terms exist. Under this constraint, we obtain the maximum likelihood estimates (MLEs) of ODE parameters and AR(1) parameters.

By plugging in the MLEs of model parameters into the likelihood, we obtain the likelihood values ${\hat{L}}_{1}$ (assuming that there are interactions) and ${\hat{L}}_{0}$ (assuming that there are no interactions), respectively. We further estimate the log-likelihood ratio,

(10) $$\mathrm{LR}=-2\log({\hat{L}}_{0}/{\hat{L}}_{1})$$

as a statistic used to test if interactions exist. By reshuffling the expression data between FGCs and somas across *n* embryos randomly, we calculate the LR value. If this permutation procedure is repeated 1000 times, we obtain the 95th percentile from 1000 LR values and use it as a critical threshold. 

### 2.8. Regression Model of Gene Networks

We view an embryo as a sample composed of FGCs and somas. Let $y_{j}\left(E_{ik}\right)$ denote the expression level of gene *j* on sample *i* from cell type *k*, expressed as a function of expression index (EI), defined as the sum of the expression values of all genes on sample *i* from cell type *k*; i.e., $E_{ik}=\overset{m}{\underset{j=1}{\sum }}y_{j}\left(E_{ik}\right)$. As a part–whole relationship, $y_{j}\left(E_{ik}\right)$ scales allometrically with $E_{ik}$ across samples, which can be described by a power equation. A regression model of gene *j* on other genes as predictors, implemented with qdODEs of Equation (3), can be formulated as

(11) $$y_{j}\left(E_{ik}\right)=P_{j}\left(E_{ik}\right)+\overset{m}{\underset{j^{\prime }=1,\;j^{\prime }\neq j}{\sum }}P_{jj\prime }\left(E_{ik}\right)+e_{j}\left(E_{ik}\right)$$

where $P_{j}\left(E_{ik}\right)$ is the independent expression component of gene *j* on sample *i* from cell type *k*; $P_{jj\prime }\left(E_{ik}\right)$ is the dependent expression component of gene *j* affected by gene *j*′; and $e_{j}\left(E_{ik}\right)$ is the residual error of gene *j*. We use a power equation to model the independent expression component and a non-parametric approach to model the dependent expression component.

If the number of genes is very large, we implement two steps for dimension reduction. The first is to use power equation-based functional clustering to classify all genes into different modules based on which genes are expressed similarly across all samples from both FGCs and somas. The second is to implement group LASSO and adaptive group LASSO to select the most significant links for each gene. After variable selection, the number of genes that are involved in the dependent component of gene *j* will reduce largely to *D_jk_*, making the full qdODEs of Equation (3) become sparse. We impose a constraint on the number of regulated genes by a regulator but no constraint on the number of regulators. By reconstructing high-dimensional but sparse networks using the sparse qdODEs, we have the capacity to identify all possible regulators. 

### 2.9. Network Recovery

Under the likelihood of the EI-varying expression of all genes, we implement the sparse qdODEs to model the mean vector of the distribution and AR(1) to model the covariance matrix. By implementing the fourth-order Runge–Kutta algorithm and (Nelder–Meade) simplex algorithm, we obtain the MLEs for all model parameters. We code $P_{j}(E_{ik})$ as nodes and $P_{jj\prime }(E_{ik})$ (*j* = 1, …, *m*; $j^{\prime }$ = 1, …, *j* − 1, *j* + 1, …, $D_{jk}$) as edges into a graph as gene networks G*_k_*($E_{ik}$), separately for FGCs and somas. These networks can capture all three possible features of gene interactions—bidirectional, weighted, and signed—because $P_{jj\prime }(E_{ik})$ can characterize the strength and sign (promotion vs. inhibition) with which gene $j^{\prime }$ affects *j* and also because $P_{jj\prime }(E_{ik})$ and $P_{j\prime j}(E_{ik})$ can describe and compare how genes *j* and $j^{\prime }$ are reciprocally affected. When compared to most existing networks that do not meet these three features simultaneously, G*_k_*($E_{ik}$) is regarded as being fully informative.

Since G*_k_*($E_{ik}$) is a function of $E_{ik}$, this suggests that we can reconstruct a network for each sample, i.e., embryo. To this end, we can compare how the networks vary structurally and functionally from one embryo to another embryo and from one stage to the next. Increasing evidence shows that a complex trait is controlled by a full set of genome-wide genes, indicating the necessity of reconstructing an omnigenic network.

### 2.10. The Coefficient of Hubness

In a network, a node with a strikingly larger number of links in comparison with other nodes is called a hub, and is believed to have a significant impact on network topology. Although hubs represent the mainstay structure of a network, nodes residing at the network periphery may play a collective role in maintaining the overall organization of the network. We propose the concept of hubness by assuming that all nodes (genes) can potentially serve as hubs, but to different extents. Links adjacent to a gene in a network include outgoing links by which the gene regulates (i.e., activate or inhibit) other genes and incoming links by which the gene is regulated by other genes. Because of its leadership role, we will only estimate the outgoing hubness of each gene. We derive a formula to calculate the strength of outgoing hubness for gene *j*, expressed as

(12) $$H_{j}=\overset{L}{\underset{j^{\prime }=1}{\sum }}\frac{\int^{\;}\left|W_{j^{\prime }\leftarrow j}\left(\times \right)\right|}{\int^{\;}W_{j^{\prime }}\left(\times \right)}\;$$

where *L* is the number of genes that are regulated by gene *j*, $W_{j^{\prime }}\left(\times \right)$ is the independent expression component of gene $j^{\prime }$, and $W_{j^{\prime }\leftarrow j}\left(\times \right)$ is the dependent expression component of gene $j^{\prime }$ affected by gene *j*. A high $H_{j}$ value is positively associated with a stronger hubness of gene *j*.

## 3. Results

### 3.1. FGC–Soma Interactions Driven by Individual Genes

**Figure 1:**
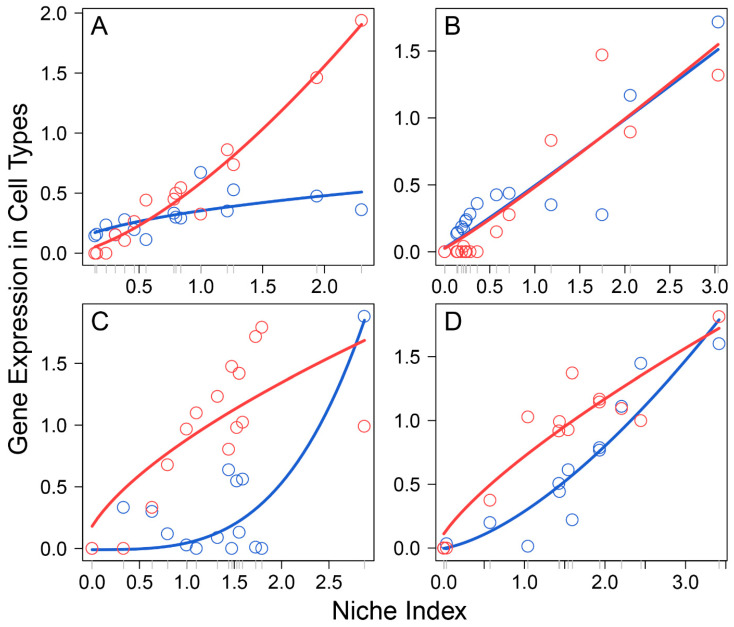
Allometric scaling of the expression level of a cell type, FGCs (blue) and somas (red), from an embryo against the NI of embryos for four randomly chosen genes, *A2M* (**A**), *ADAP2* (**B**), *AKR1B10* (**C**), and *ANXA1* (**D**). Each dot denotes the value of gene expression on a cell type from an embryo, and lines present the fitting of a power equation to the data.

Li et al.’s data [3] comprised 17 female embryos sampled at a range of developmental stages from 4 to 26 weeks post-fertilization, which formed an early stage of primordial folliculogenesis. In each embryo, multiple subpopulations of cells were isolated to profile the transcriptomes of FGCs and somas by single-cell RNA-seq, obtaining a total of 1220 genes that are expressed jointly between the two interactive cell types. We took the expression mean of each gene over all cell subpopulations from each cell type, from which to calculate the NI of the gene on each embryo. By plotting the expression of each gene on each cell type against the NI across embryos, we found that this relationship can be reasonably fitted by a power equation (*p* < 0.05; Figure S1), although the form of curve fitting varied among genes and between cell type for the same gene (Figure 1). Thus, the power equation was used to model the independent expression component of Equation (2)’s qdODEs, but with the dependent expression component modelled by an LOP-based nonparametric approach. By estimating the independent and dependent expression component integrals of the same gene expressed on FGCs and somas, we characterized how the two cell types interact with each other, driven by individual genes.

**Figure 2:**
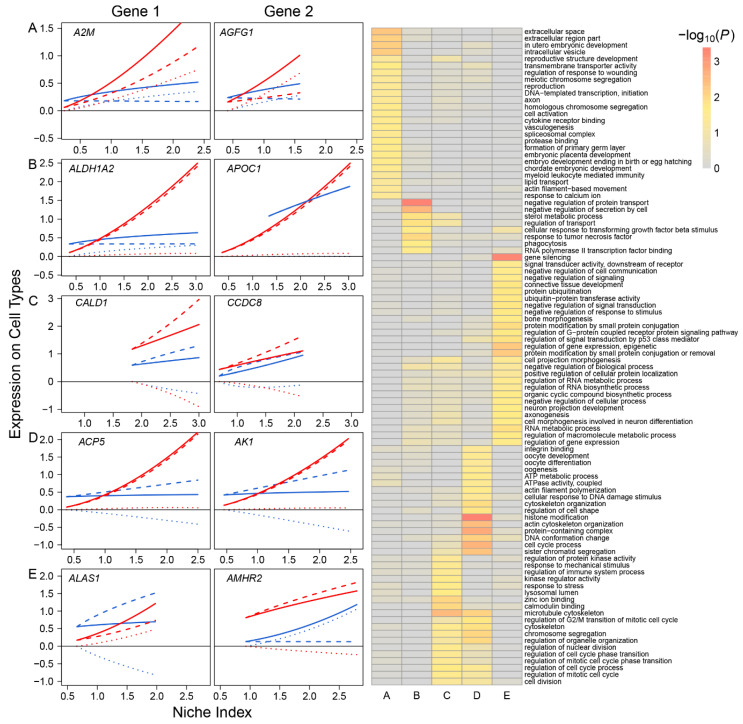
Five different types of FGC–soma interactions driven by genes. **Left panel**: The NI-varying change of each interaction type, illustrated by two representative genes. (**A**) Synergism—FGCs and the somas activate each other, driven by 785 genes. (**B**) Directional synergism—one cell type activates the other whereas the second has no impact on the first, driven by 82 genes. (**C**) Antagonism—both cell types inhibit each other, driven by 52 genes. (**D**) Directional antagonism—one cell type inhibits the other whereas the second has no impact on the first, driven by 87 genes. (**E**) Altruism/exploitation—one cell type activates (or inhibits) the other but the second reversely inhibits (or activates) the first, driven by 214 genes. For each interaction type, solid, slashed, and dotted lines denote the overall expression profile, independent expression component profile, and dependent expression component profile of each cell type, FGCs (blue) and somas (red). **Right panel**: GO analysis of genes that drive various types of interactions (**A**–**E**).

Based on the pattern of how they impact FGC–soma interactions, we classified all participating genes into five different types: *synergistic regulators*, including 785 genes, such as *A2M* and *AGFG1*, through which FGCs and somas activate each other (Figure 2A); *directional synergistic regulators*, including 82 genes, such as *ALDH1A2*, by which the somas activate FGCs but the FGCs are neutral to the somas, and *APOC1*, by which FGCs activate the somas but the somas are neutral to FGCs (Figure 2B); *antagonistic regulators*, including 52 genes, such as *CALD1* and *CCDC8*, by which FGCs and the somas inhibit each other (Figure 2C); *directional antagonistic regulators*, including 87 genes, such as *ACP5*, by which somas inhibit FGCs but FGCs are neutral to the somas, and *AK1*, by which FGCs inhibit the somas but the somas are neutral to FGCs (Figure 2D); and *altruistic regulators*, including 214 genes, such as *ALAS1*, by which FGCs activate the somas but the somas inhibit FGCs, and *AMHR2*, by which the somas activate FGCs but FGCs inhibit somas (Figure 2E). Altruistic genes regulate the altruism of cell type 1 toward cell type 2, which can also be explained as *exploitation regulators* since they regulate the predation of cell type 2 to cell type 1. Gene enrichment analysis shows that these five types of genes display distinct biological functions (right panel, Figure 2); for example, synergistic regulators mostly regulate intracellular space and vesicle, meiotic chromosome segregation, and embryo development; antagonistic regulators are associated with cell division, cell cycle, cell mitotic process and response to stress; and altruistic regulators regulate gene and epigenetic expression, RNA biosynthetic process, RNA metabolic process, signal transduction, and cell communication.

### 3.2. Gene Regulatory Networks Mediating the Coordinated Development of FGCs and Somas

**Figure 3:**
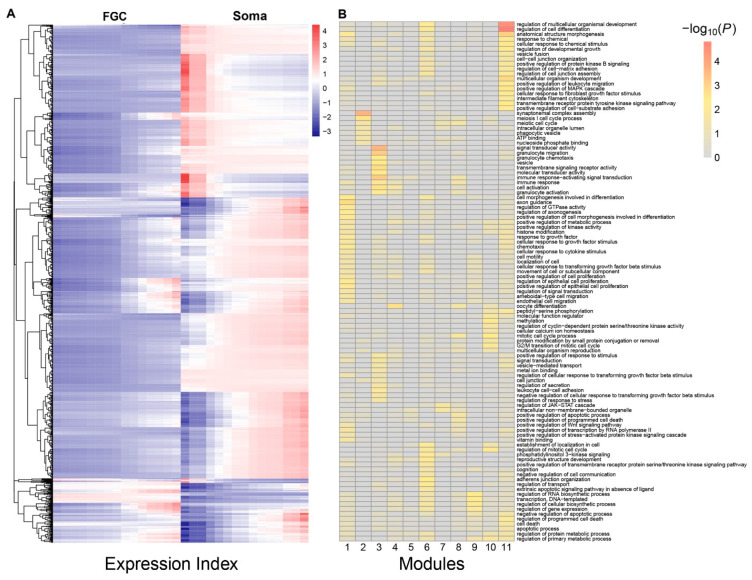
Functional clustering of genes into 11 distinct modules based on the allometric scaling pattern of the expression of individual genes with EI across FGC and soma samples. (**A**) The heat map of differentiated gene expression by functional clustering. (**B**) GO analysis of genes from modules 1–11.

Multiple genes are organized into a network to mediate FGC–soma interactions and development. To reconstruct gene regulatory networks that guide how FGCs communicate with somas, we assume functional modularity. That is, genes function differently, but seem to be organized into common functional groups (modules), thus causing the gene network to exhibit modularity. Genes from the same module display a more similar function than those from different modules. The power equation was found to well fit the dynamic trajectories of how genes are expressed differently with EI across samples (Figure S2). We implemented power equation-based functional clustering to classify all genes into different modules. Using AIC values, we identified 11 as the optimal number of modules (Figure 3A). Gene enrichment analysis suggested that genes in each module displayed distinct patterns of biological function (Figure 3B).

**Figure 4:**
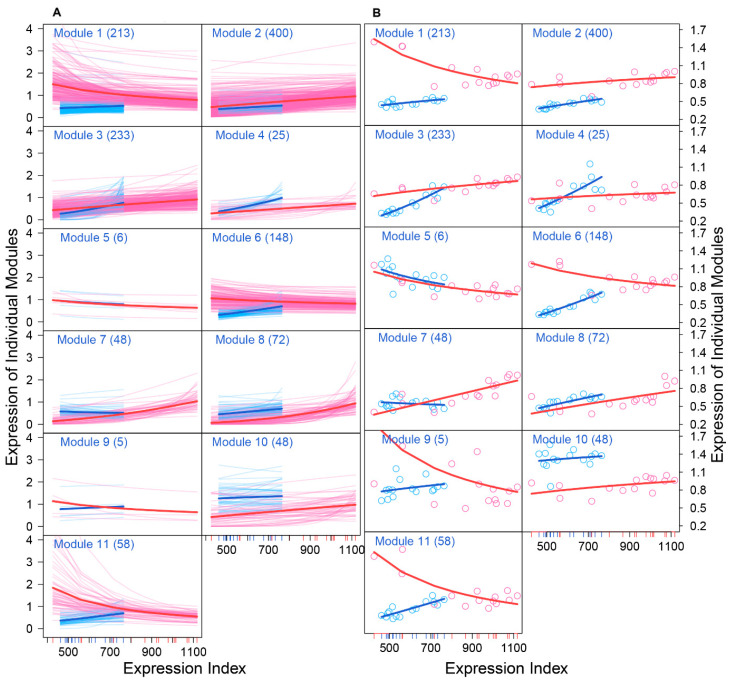
Distinct EI-varying pattern of 11 modules separately for FGCs (blue) and somas (red). (**A**) The change of expression of all genes within a module across EI. Thin lines denote the expression profiles of individual genes and thick lines are the average expression profiles of all genes from each module. (**B**) The fitness of averaged expression values of all genes from each module across EI. Each dot denotes the averaged expression value of genes on each sample.

We drew the EI-varying expression profiles of genes from each module, separately for FGCs and somas (Figure 4). As the total amount of gene expression, EI reflects the carrying capacity of a sample to undertake its function. Somas span a wide spectrum of EI across samples, at a lower part of which FGCs reside, suggesting that somas tend to reserve richer resources used to maintain FGCs’ function (Figure 4A). Expression of genes in almost all modules consistently increased as EI increased in the case of FGCs, but more often decreased in the case of somas. All modules, except for module 5, displayed different amounts and patterns of EI-varying expression profiles between FGCs and somas. Overall, modules 1, 6, 9, and 11 had a much larger amount of expression on somas than FGCs and were expressed decreasingly with EI on somas but increasingly with EI on FGCs (Figure 4B). Taken together, the spectrum of EI and the EI-varying expression pattern of modules can be used to distinguish between fetal germlines and somas.

**Figure 5:**
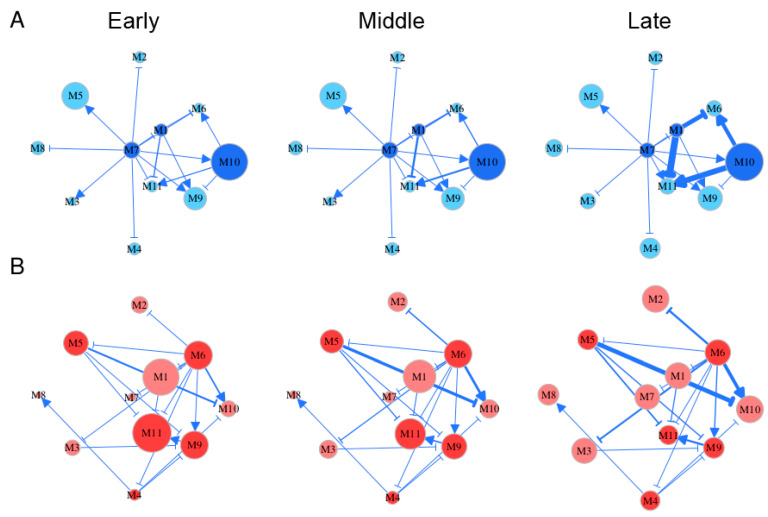
Stage-specific (early, middle, and late) gene networks among 11 modules, labeled M1–M11, for FGCs (blue) (**A**) and soma (red) (**B**). The size of circles is proportional to the mean expression level of all genes from a module. Arrowed lines and T-shaped lines denote the activation and inhibition of one module on the second, respectively. Hub modules in FGC and soma networks are highlighted in dark blue and dark red, respectively.

Embryo development spans several distinct stages during which gene regulation may vary. By roughly classifying Li et al.’s time-varying samples [3] into early (5–10 weeks post-fertilization), middle (11–14 weeks post-fertilization), and late stages (18–26 weeks post-fertilization), we reconstructed stage-specific gene regulatory networks at the module level for FGCs and somas, respectively (Figure 5). From these networks, we characterized how the structure and organization of module interaction changed over developmental time and differed between the two cell types. Both FGC and soma networks were quite sparse, containing links indicative of only directional synergism and directional antagonism, suggesting the role of asymmetric interactions observed in macroscopic organisms [30] is also present in mediating gene communities. However, these two types of networks differ remarkably in topological structure. The most striking difference lay in hub modules, thought to play a central role in network structure. Despite its low expression on FGCs, module 7 served as the primary hub that exhibited outgoing links with all other modules (Figure 5A). The secondary hubs in the FGC network were modules 1 and 10, with the latter being heavily expressed. Module 7 participated in cell apoptosis by regulating JAK-STAT cascades, module 1 regulated the Wnt signaling pathway implicated in stem cell control, and module 10 controlled mitotic behavior and methylation. These three hubs played an essential role in coordinating the development of fetal germlines by culling out inferior FGCs (module 7) and proliferating superior FGCs (modules 1 and 10).

Both modules 6 and 11 in the FGC network received incoming links, thus were regarded as target modules. These two modules participated in a wide range of activities; i.e., module 6 regulated mitotic cell cycle, reproductive structure development, adherens junction organization, and the extrinsic apoptotic signaling pathway, and module 11 regulated cell apoptosis, cell junction assembly, cell-matrix adhesion, MAPK cascade, and the transmembrane receptor protein tyrosine kinase signaling pathway. All these activities are crucial for the formation of primordial follicles. It was interesting to see that module 6 became the primary hub in the soma network that outgoing links to almost all other modules mostly through inhibition (Figure 5B). The position change of versatile module 6 from a target of FGCs to a regulator of somas may not only bridge a functional link between these two cell types, but also imply that FGCs play a leadership role in follicle development by regulating somas. Unlike module 6, module 11 still served as a target in the soma network. Module 11 did not only help FGCs select superior cells, but also regulated the cell–matrix adhesion and transmembrane of somas, facilitating follicle formation. 

Gene networks underlying each cell type changed quantitatively rather than qualitatively during different stages of embryo development; i.e., gene–gene interactions displayed a stage-dependent change in their strength rather than their causality and sign (Figure 5). For example, in the FGC network, the structural role of modules 7, 1, and 11 did not change from stage 1 to 3, but the strength at which module 1 inhibited 6 and 11 and the strength at which module 10 activated 6 and 10 increased slightly from stage 1 to 2 but dramatically from stage 2 to 3. A small network constituted by modules 1, 6, 10, and 11 was a key driver of stage-specific change of the FGC network, in which modules 6 and 11 merely received incoming links. Time-dependent change in the soma network was found in the strength at which module 9 activated module 11, the strength at which module 5 inhibited 10 and 11, and the strength at which module 6 activated 10. Unlike the FGC network, where the independent expression level of each module (except for 4) was quite stable over time, the independent expression of many modules in the soma network changed dramatically from stage to stage; e.g., module 7 increased gradually but module 11 decreased gradually. Both modules 4 and 10 increased their independent expression level in both networks, especially from stage 2 to 3. Gene enrichment analysis showed that these two modules contained genes that participate in oocyte differentiation (Figure 3), suggesting that both FGC and soma networks have been well organized for primordial follicles to develop after FGCs enter the oogenesis stage. In addition, module 10 was found to be functionally related to mitotic cycles and cell proliferation, from which we postulate that the development of oogonia requires nutritional support from an increasingly growing number of somas.

**Figure 6:**
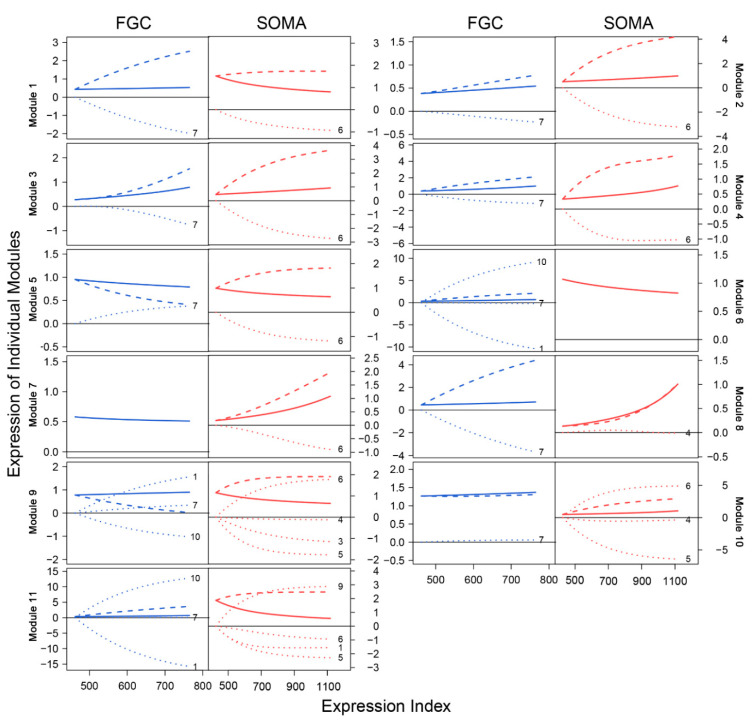
EI-varying changes of the overall gene expression of each module (solid line) and its independent (slash line) and dependent expression components (dot line) for FGCs (blue) and somas (red).

We characterized how genes of different modules were expressed differently over EI (Figure 6). We decomposed the overall gene expression profile of each module into its independent expression component and dependent component. The pattern of gene expression differed dramatically between FGCs and somas. In the FGC network, all modules were regulated by hub module 7, whereas in the soma network, all modules, except for 8, were regulated by hub module 6. The overall expression level of module 1 slightly increased with EI in the FGC but decreased in somas, yet genes in this module virtually increased their expression dramatically in both cell types if these genes were not inhibited by modules 7 and 6, respectively. The expression profiles of most modules were strikingly affected by these two hub modules (Figure 6). As a hub in the soma network, module 6 in the FGC network became a target, largely activated by module 10 and slightly by module 7 but largely inhibited by module 1, leading the overall expression level of module 6 to be similar to its independent expression level. Module 6 in the soma network only affected other modules, thus leading its observed expression level to be equal to its independent expression level. Although the independent expression curve of module 6 and its observed expression curve overlapped in both FGC and soma networks, the underlying reasons that cause such consistency are cell-type dependent.

### 3.3. Genes Mediating Signaling Pathways across Different Cell Types and from Mitosis to Meiosis

**Figure 7:**
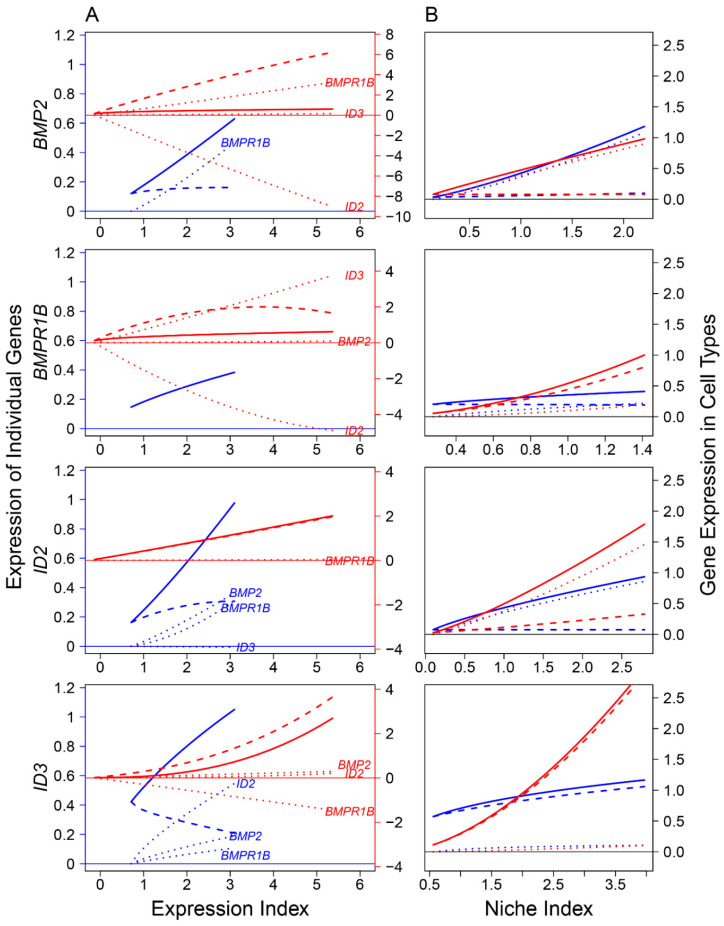
FGC–soma interactions driven by genes involved in the bone morphogenic protein (BMP) signaling pathway, where *BMP2* is the ligand highly expressed in somas, *BMPR1B* is the receptor expressed in both somas and FGCs, and *ID2* and *ID3* are the targets specifically upregulated in RA-responsive, meiotic prophase, and oogenesis FGCs. (**A**) *BMP2*, *BMPR1B*, *ID2*, and *ID3* worked together in a network to mediate FGC and soma development as a function of expression index, respectively. Left and right sides of the figure denote the expression levels of genes in FGCs (blue) and somas (red), respectively. (**B**) The niche index-varying pattern of interactions between FGCs (blue) and somas (red) driven by individual genes. Solid thick lines, slashed lines, and dotted lines denote the overall expression level, independent expression level, and dependent expression level, respectively.

**Figure 8:**
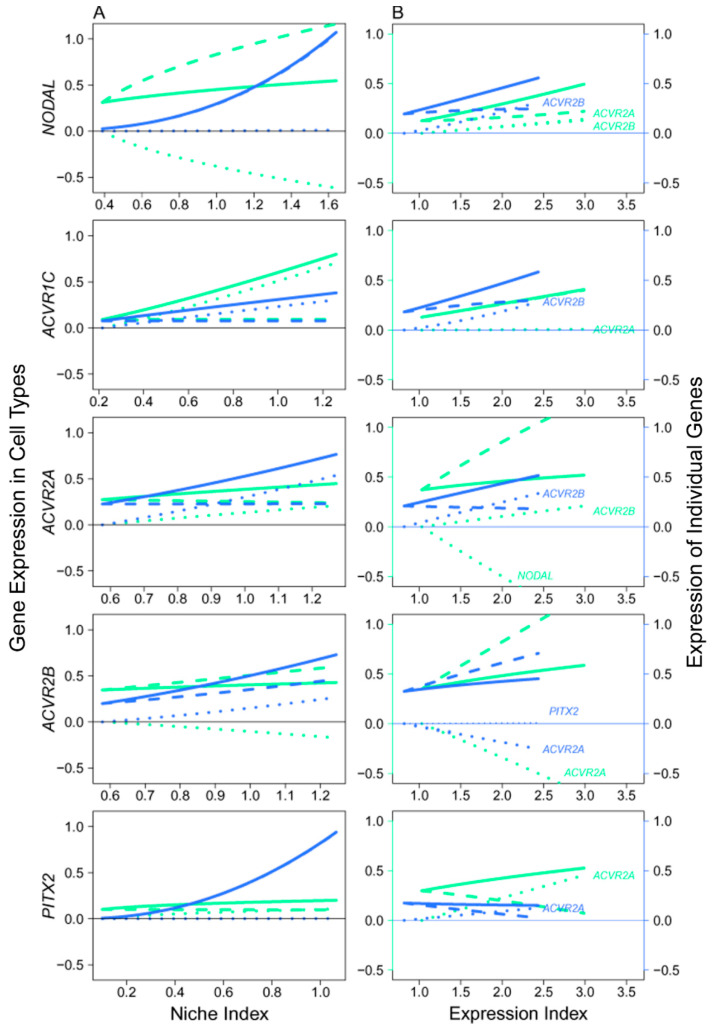
Mitotic–meiotic interactions driven by genes involved in the NODAL signaling pathway, where *NODAL* is the ligand expressed in mitotic FGCs, *ACVR1C*, *ACVR1A*, and *ACVR1B* are the receptors and *PITX2* is the target, with both receptors and target being specifically expressed in meiotic FGCs. (**A**) The niche index-varying pattern of interactions between mitotic FGCs (green) and meiotic FGCs (light blue) driven by individual genes. Solid thick lines, slash lines, and dots lines denote the overall expression level, independent expression level, and dependent expression level, respectively. (**B**) *NODAL*, *ACVR1A*, *ACVR1B*, *ACVR1C*, and *PITX2* worked together in a network to mediate mitotic and meiotic phases of FGC development as a function of expression index, respectively. Left and right sides of the figure denote the expression levels of genes in mitotic FGCs (green) and meiotic FGCs (light blue).

**Figure 9:**
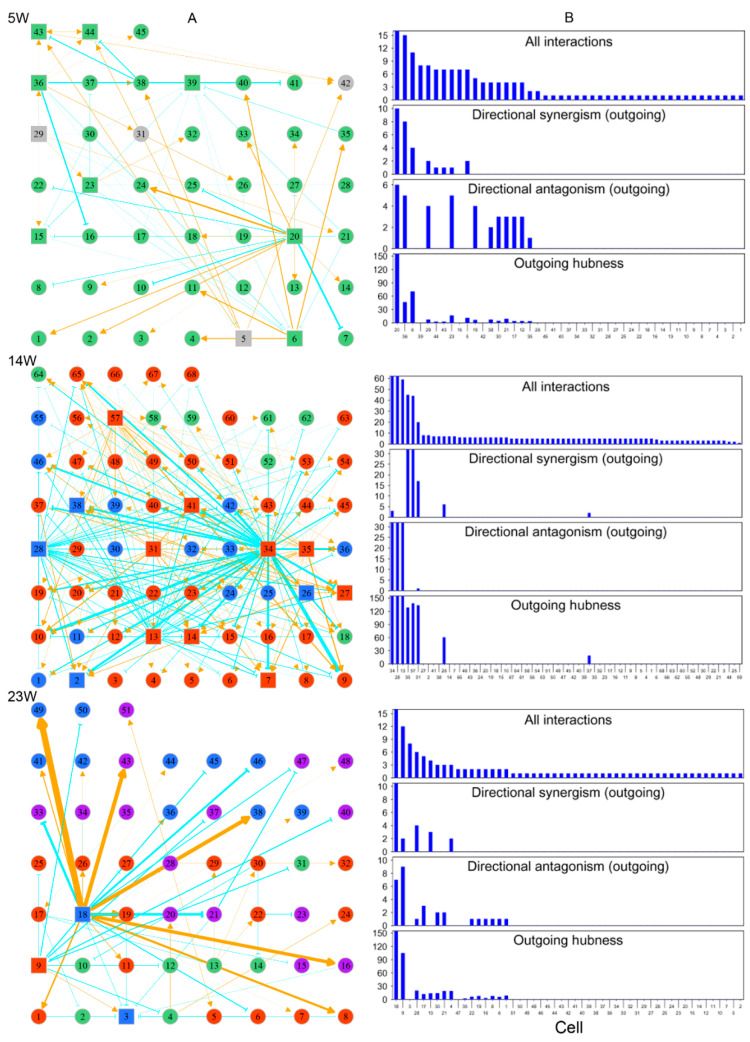
Networks of cell–cell interactions, guided by genes, at three different stages of embryo development (5, 14, and 23 weeks post-fertilization). (**A**) Topologies of time series of networks among randomly sampled cells. Cells in different FGC phases (determined by biomarkers) denoted with colored circles or squares (standing for hubs): green for mitosis, dark red for RA-responsive phase, light blue for meiosis, and purple for oogenesis. Gray color indicates cells whose phase cannot be determined. Yellow-orange arrowed lines and blue-green T-shaped lines denote promotion and inhibition, respectively. (**B**) The distribution of the number of all interactions, outgoing directional synergism, and outgoing directional antagonism and outgoing hubness across FGCs within the 5-week, 14-week and 23-week networks.

Previous studies identified the role of several signaling pathways, including bone morphogenic protein (BMP) and NOTCH, in mediating FGC development at various stages [3,31,32,33]. The proper development of FGCs is determined directly by their coding genes, whose expression is activated by ligand-receptor binding affinities that transduce signals. We reconstructed ligand-receptor-target regulatory networks to investigate how signaling pathways affect FGC–soma interactions across embryos (Figure 7, Figure 8 and Figure 9). We found that FGCs and somatic cells were transcriptionally linked by the reciprocal transduction of signals. Types and strengths of FGC–soma interactions mediated by signaling pathways vary dynamically over embryo development. For the BMP pathway on FGCs, targets *ID2* and *ID3* were activated by ligand *BMP2* and receptor *BMPR1B*, making the overall expression level of the former largely beyond their independent level (Figure 7A). Downstream *ID3* was strikingly promoted not only by *BMP2* and *BMPR1B* but also by *ID2*, although *ID2* received a small feedback from *ID3*. Ligand *BMP2* received a strong feedback from receptor *BMPR1B*, but surprisingly, *BMPR1B* was only slightly regulated by *BMP2*, rather affected strongly by *ID2* (inhibition) and *ID3* (promotion). Both *BMP2* and *BMPR1B* on somatic cells were remarkably affected by targets, and it is interesting to see that all ligands, receptors, and targets promoted the mutual synergism between FGC and somatic cells (Figure 7B).

The FGC–soma interaction is also mediated by the NOTCH signaling pathway, but in different ways. In the gene networks reconstructed for the NOTCH pathway (Figure S3), we found that FGCs were inhibited by somas whereas somas were promoted by FGCs at receptor *NOTCH2*. Both *DLL3* and *JAG1* are ligands on FGCs, but through them FGCs were affected by somas differently. FGCs were inhibited by somas at *DLL3* but promoted at *JAG1* (Figure S3). We postulate that these differences may lead to the augmented expression of target *HES1* on somas over FGCs. In the end, the role of genes in the NOTCH signaling pathway was modulated by their regulatory networks (Figure S3). 

We analyzed the NODAL signaling pathway that transduces signals from the mitotic phase of FGCs to their meiotic phase [3]. The embryos after 14 w post-fertilization started to develop meiotic FGCs, thus we took the expression means of each gene (involved in the NODAL pathway) over all FGCs in mitotic and meiotic phases, respectively, for all embryos older than this age. The qdODE game model was used to characterize how mitotic cells interact with meiotic cells through these genes, providing a clue to map the developmental trajectories of FGCs from early to late phases. 

In the NODAL pathway, ligand *NODAL* functions in mitotic FGCs, whereas receptors *ACVR1C*, *ACVR1A*, and *ACVR1B* and target *PITX2 function* in meiotic FGCs, suggesting that late-phase FGCs receive NODAL signals secreted from those in early phases [3]. However, a detailed insight into the regulatory mechanisms underlying this process has not been established. Results from the qdODE game model show that *NODAL* expressed in mitotic FGCs had no impact on *NODAL* expressed in meiotic FGCs, while meiotically expressed *NODAL* exerted a strong negative regulation on mitotically expressed *NODAL* (Figure 8A). Three receptor genes helped early- and late-phase FGCs establish a synergistic relationship, showing their direct role in mediating the proper development of FGCs. We found that the expression of the target gene in meiotic FGCs was not affected by the expression of mitotic FGCs. Figure 8B illustrates how the receptors and target interacted with each other to affect mitotic and meiotic FGCs, respectively. In mitotic FGCs, the expression of *NODAL* was promoted by both *ACVR1A* and *ACVR1B, whereas ACVR1A* inhibiting *ACVR1B was heavily inhibited by NODAL. In meiotic FGCs, NODAL was also promoted by receptors, but the receptors generally received promotion from each other and from the target.*

### 3.4. Modeling Cell Heterogeneity and Interactions within Embryos

Each FGC can potentially develop toward an oogonia, but this process is subject to strict natural selection through competition and cooperation. We reconstructed three cell–cell rewiring networks, respectively, at 5 weeks, 14 weeks, and 23 weeks post-fertilization (Figure 9) in order to reveal how cells cooperate or compete against each other under the overall guidance of all genes. We randomly chose an embryo from each of three distinct stages, 5 weeks, 14 weeks, and 23 weeks post-fertilization, and obtained 45, 68, and 51 FGCs from these three embryos, respectively. The phase of an FGC was determined by a set of phase-specific biomarkers [3]. Using these FGCs, we reconstructed cell–cell game networks for early, middle, and late embryos with FGCs in different phases, which helped us to omnidirectionally explore the general pattern of FGC–FGC heterogeneity and interactions. 

By plotting the gene expression of individual cells against the EI of genes, we found the existence of allometric relationships by the power equation (Figure S4). We found that all three networks were composed of directional synergism and directional antagonism; these asymmetric interactions are similar to those observed in macroscopic organisms [30]. The networks at different stages of embryo development displayed different features. At 5 w post-fertilization, the 45-node network inferred was mainly composed of the interactions among FGCs which were in the mitotic phase (Figure 9A). This network was found to be quite sparse, with structure determined by two (4%) primary hubs that regulated other cells through both activation and inhibition, 15 (33%) secondary hubs that regulated and also are regulated by other cells, and the remaining 28 (63%) nodes that are only regulated by other cells. The 68-node network at 14 w post-fertilization captured interactions among 7 (10%) FGCs in the mitotic phase, 45 (66%) FGCs in the RA signaling-responsive phase, and 16 (24%) FGCs in the meiotic phases (Figure 9B). As compared to the 5-w network, this network showed increasing topological complexity, but only approximately six (8%) cells served as hubs that either activated or inhibited other cells, with a majority of cells (92%) being subordinates (Figure 9B). Three directional synergism hubs, 31, 35, and 57, were all in the RA signaling-responsive phase, on average regulating 2, 14, and 6 FGCs in mitotic, RA signaling-responsive, and meiotic phases, respectively. Of three directional antagonism hubs, FGCs 13 and 34 in the RA signaling-responsive phase, on average, regulated 6, 40, and 14 FGCs in the mitotic, RA signaling-responsive, and meiotic phases, respectively, and FGC 28 in the meiotic phase regulated 6, 42, and 14 FGCs in the mitotic, RA signaling-responsive, and meiotic phases, respectively. 

The 51-node network at 23 w post-fertilization included interactions among 7 (14%) FGCs in the mitotic phase, 17 (33%) in the RA signaling-responsive phase, 12 (24%) in the meiotic phases, and 15 (29%) in the oogenesis phase (Figure 9A). Relative to the 14-w network, this network was dominated by three hub cells (5%), one primary hub cell 18 in the meiotic phase and two secondary hub cells 3 and 9 in RA signaling-responsive and meiotic phases. The primary hub rarely regulated FGCs that underwent the mitotic phase, but exerted an increasing influence on FGCs in the RA signaling-responsive phase. It triggered a tremendous impact on FGCs that were experiencing the same meiotic phase and the subsequent oogenetic phase.

Taken together, cell networks follow a similar topological structure dominated by directional synergism and directional antagonism. Network structure and organization changed dramatically in response to embryo development. The network at an early stage of embryo development was sparse, in which about 37% of mitotic FGCs served as hubs to maintain network stability. As embryos developed, FGCs at different stages co-occurred to form more complex networks, but these networks were often mediated by a much smaller set of leaders (5–8%). The number of hubs with a strong leadership (quantified by the coefficient of outgoing hubness) proportionally decreased with embryo development (Figure 9B). We speculate that the development-dependent decrease of hub proportion was the consequence of natural selection by which a small portion of germ cells grew vitally at the cost of a majority of subordinate and gradually culled cells.

### 3.5. Computer Simulation

To investigate the statistical properties of our qdODE game model, we performed computer simulation by mimicking Li et al.’s [3] sampling strategy. For each type of interaction, we simulated expression values of a gene under two different sample sizes (15 and 50 embryos), respectively, using qdODE parameters and residual variances estimated from Li et al.’s data. In general, the qdODE game model reasonably estimated the general trends of NI-varying gene expression including independent expression and dependent expression components when sample size was 15 (Figure S5A). The estimation accuracy of gene expression profiles increased dramatically when sample size increased to 50 (Figure S5B). We also simulated the expression data by reducing the residual variance, which, as expected, increased estimation precision. In practice, if measurement errors cannot be well controlled due to technical issues, we could still reach the expected precision of parameter estimation by increasing sample size.

We performed an additional simulation study to justify the need of the qdODE game model. Our game model characterizes NI-varying gene expression changes composed of independent and dependent components, whereas a traditional model only includes the independent component, which we call the non-game model. We used game and non-game models to simulate the expression data of genes under a sample size of 15 and then used both models to reciprocally analyze these two datasets. As expected, the game model well estimated NI-varying expression curves for the data simulated under the game model, but the non-game model provided a biased estimate of expression curves for the same data (Figure S6). This suggests that for data that contains interaction components, the traditional non-game model does not perform well. To analyze this type of data, the game model should be used. On the other hand, we found that both models can provide reasonable estimates of expression curves from the data that were simulated by either the game model or non-game model (Figure S6). Taken together, as the generalization of the non-game model, the game model can be used to analyze any type of data.

## 4. Discussion

For this article, we developed and applied the so-called qdODE game model to infer the gene regulatory networks of primordial follicle development. The model can chart a comprehensive atlas for the genomic signatures of cell interactions and crosstalk across fetal germlines and their microenvironment, which not only supports the known signaling pathways that link FGCs and their gonadal niche cells [34,35,36], but also characterizes several previously unknown mechanisms behind cell heterogeneity, development, and evolution. To our knowledge, the qdODE game model presents the very first theory of its kind that can systematically reconstruct the genomic landscapes that guide, orchestrate, and impact FGC–soma interactions. We classified all participating genes into five categories according to their patterns of impact; i.e., synergistic genes that favor the mutualism of the two cell types, antagonistic genes that damage their mutual cooperation, directionally synergistic genes that support the unidirectional payout of one cell type with the other, directionally antagonistic genes that condition the unidirectional predation of one cell type with the other, and altruistic or exploitative genes that allow one cell type to benefit at the cost of the counterpart. Our qdODE game model not only qualitatively discerned each of these patterns, but also quantitatively estimated their strengths.

By analyzing the scRNA-seq data of human primordial follicles [3], we found that two-thirds of genes promoted the synergism of FGCs and somas. From an evolutionary viewpoint, cell–cell cooperation as a process in which two parties work or act together for mutual benefits helps to increase the “fitness” of the whole system constituted by the two parties [37]. The establishment of a comprehensive encyclopedia recording the role of each gene will shed light on the precise mechanisms underlying FGC and gonadal somatic cell development, facilitating the identification of FGC-like cell differentiation in vitro.

The qdODE game model can reconstruct detailed gene regulatory networks for the developmental trajectories of FGCs and somas. These networks that are dynamic, but inferred from static data, can track and visualize how the topological structure of gene–gene interactions change dynamically during fetal development. These cell-type specific gene networks allow the question of how the networks differently impact FGCs and somatic cell growth to be addressed. The FGC networks are qualitatively different from the soma networks in terms of the type of hubs and their links with other genes or modules, but both networks change quantitatively in interaction strength with different stages of embryo development. These qualitative and quantitative discrepancies may provide unique clues that can help interpret the genomic mechanisms that drive cell type-specific growth and cell–cell interactions across fetal germs and somas.

In analyzing primordial follicle data provided by Li et al. [3], we identified key genes or modules that determine the predominant role of FGCs over somas required for the normal development of primordial follicles. As a target gene of the FGC network that mediates biological processes directly related to FGC growth, module 6 becomes an important hub in the soma network to regulate many other genes (Figure 5). Thus, we postulate that genes in module 6 determine different roles of FGCs and somas in follicle development. As seen by GO enrichment analysis, this module contains genes that determine apoptosis, cell proliferation, and cell–cell communication, showing its critical roles in mediating the selection of superior FGCs and the proliferation of somas.

Human embryos are characterized by high cellular heterogeneity thought to be evolutionarily advantageous [38]. Heterogeneity and diversity are a widespread phenomenon by which organisms can better adapt to a perturbed environment and, thereby, evolve into a better form and function. Female fetal germs involve four cell subpopulations, mitotic, RA signaling-responsive, meiotic, and oogenesis, that exist at different developmental stages, whereas their niche cells contain four subpopulations, endothelial, early granulosa, mural granulosa, and late granulosa [3]. In embryos at an early stage of development (e.g., 5 weeks post-fertilization), only mitotic cells may exist, and the other cell subpopulations emerge gradually with embryo development. Using single-cell analysis, Li et al. [3] found the heterogeneity of gene expression profiles among individual FGCs within and between subpopulations.

The qdODE game model can characterize how functionally and developmentally different cells communicate with each other in a network and understand gene-driven cell-to-cell communications within and between phase-specific subpopulations from the same embryo (Figure 9). Through network analysis, we found that the degree of cellular heterogeneity increased with embryo development, leading superior FGCs to be more superior and inferior ones to be more inferior. In the end, only a small portion of FGCs can successfully become the hubs of cell networks and finally develop into oocytes. Coordinated cell interactions and rewiring are essential for proper organ development [39]. By inferring the cellular networks, we can evaluate and identify which cells play a readership role in regulating cell–cell interactions as hubs at each stage of embryo development.

Understanding genomic machineries behind the developmental variation and interactions of primordial follicles is one of the major tasks in reproductive biology and medicine. Not relying on temporal data, the qdODE game model derived from the integration of allometric scaling theory and evolutionary game theory, whose mathematical properties have been extensively studied [40], can infer and recover developmental trajectories of FGC–soma interactions and gene networks from steady-state expression data. Given that FGC development is regulated by epigenetic reprogramming [41] and involves an assembly of large RNA-protein granules [42], it is crucial to modify our qdODE game model as a tool to reconstruct epigenetic regulatory networks. With this modification, we will be in an excellent position to understand primordial follicle development at unprecedented resolution.

## 5. Conclusions

The proper development of primordial follicles towards maturing and eventually releasing the oocyte for potential fertilization critically depends on the coordinated interactions between cells from these two components. Using advanced single-cell analysis techniques, many studies have demonstrated the fundamental importance of gene regulator networks in mediating germline–soma interactions. However, existing approaches for network reconstruction do not consider the asynchronous and heterogeneous nature of follicle development, limiting our precise understanding of the genomic mechanisms by which germlines and somas establish communication and crosstalk. More powerful approaches are sorely needed to identify gene networks that are biologically more meaningful for germ cell development. In particular, these approaches can take advantage of increasingly available single-cell data collected from primordial follicles at unprecedented resolution.

Our model can reveal the genomic machineries of primordial follicles by charting a complete atlas of the role of each gene in modulating germline–soma interactions, their pattern, strength, and dynamics. The unique capacity of our model results from the seamless integration of allometric scaling theory, widely used to explain biological diversity in ecological research, and evolutionary game theory, a theory that studies the dynamic change of interaction strategies by individual players. By applying our model to single-cell RNA-seq data collected from more than a thousand cells in the formation of primordial follicles, we obtained several previously uncharacterized discoveries regarding follicle development:

- We created a comprehensive encyclopedia of distinct roles played by each gene in germline–soma communications. We found that about 70% of genes mediate the synergistic relationship between fetal germ cells and somas.
- We reconstructed fully informative, cell type-specific gene regulatory networks from which key hub genes or modules are detected to distinguish germline development from soma development.
- We identified specific genes that guide the response of late-phase germ cells to signaling pathways secreted from early-phase germ cells.
- We recovered cellular interaction networks of germlines from the same embryo, providing new insight into how cell heterogeneity operates as an evolutionary force to select superior oocytes for female fertility.

As compared to widely used network approaches, such as Cytoscape [43], the technical elegance of our model includes its capacity to reconstruct informative, dynamic, omnidirectional, and context-specific networks from static data. Traditionally, the inference of such meaningful networks critically depends on high-density temporal data. Given the unavailability of such expensive data in embryology and other biological fields, our model may readily find valuable use in a wide range of biological and biomedical disciplines. In summary, our model presents a conceptual and methodological advance that facilitates the effective and efficient analysis of single-cell data in a quest to unravel the genomic mechanisms of follicle development. It provides a powerful means for reproductive biologists and medical professionals to extract and excavate general principles that underlie female fertility.

## Figures and Tables

**Table 1:** Qualitative class of FGC–soma interaction and its quantitative characterization by the qdODE game model.

No.	Quantitative Description	Qualitative Definition		
		$\;P_{k\leftarrow k^{\prime }j}\left(N_{ij}\right)$		$\;\;P_{k^{\prime }\leftarrow kj}\left(N_{ij}\right)\;\;$
1	Symmetric synergism	+	=	+
2	Asymmetric synergism	+	≠	+
3	Directional synergism toward *k*	+		0
4	Directional synergism toward *k*′	0		+
5	Altruism toward *k* or exploitation by *k*	+		−
6	Altruism toward *k*′ or exploitation by *k*′	−		+
7	Symmetric antagonism	−	=	−
8	Asymmetric antagonism	−	≠	−
9	Directional antagonism toward *k*	−		0
10	Directional antagonism toward *k*′	0		−
11	Coexistence	0		0

Note:$\;P_{k\leftarrow k^{\prime }j}\left(N_{ij}\right)$ and $P_{k^{\prime }\leftarrow kj}\left(N_{ij}\right)$ are the dependent expression levels of cell type *k* by cell type *k’* and cell type *k’* by cell type *k*, respectively.

## Data Availability

The data and code uploaded at https://github.com/ccbqwang/FS can be freely uploaded and used by researchers worldwide. They can also be requested from the corresponding author.

## Supplementary Materials

The following are available online at https://www.mdpi.com/article/10.3390/cells11030332/s1, Figure S1: Plots of residuals from the fitting of the power equation vs. predicted expression values for four randomly chosen genes, *A2M* (A), *ADAP2* (B), *AKR1B10* (C), and *ANXA1* (D), for FGCs (red) and somas (blue). The ticks at the x-axis indicate the nicheindices of embryos; Figure S2: (A) Fitting of power equation to the expression of individual genes against expression indicesfor FGCs (blue) and somas (red) across embryos. (B) Plots of residuals from the fitting of the power equation vs. predicted expression values. As an example, we randomly chose genes, *A2M* (a), *ADAP2* (b), *AKR1B10* (c), and *ANXA* (d).The ticks at the x-axis indicate the expression indices of FGCs or somas: Figure S3: FGC-soma interactions driven by genes involved in the NOTCH signaling pathway, where DLL3 is the ligand highly expressed in all phases of FGCs and *JAG1* is the ligand specifically expressed in oogenesis phase FGCs, *NOTCH2* is the receptor and *HES1* is the target, both of which are highly expressed in nearly all FGCs. (A) *DLL3*, *JAG1*, *NOTCH2*, and *HES1* work together in a network to mediate FGCand soma development as a function of expression index, respectively. Left and right sides of the figure denote the expression levels of genes in FGCs (blue) and the somas (red), respectively. (B) The niche index-varying pattern of interactions between FGCs (blue) and somas (red) driven by individual genes. Solid thick lines, slash lines, and dots lines denote the overall expression level, independent expression level, and dependent expression level, respectively. Ligand *DLL3* is expressed both in FGCs and somatic cells, suggesting that it provides a foundation for NOTCH signaling interaction between these two different types of cells. Our qdODE model can dissect how *DDL3* determine FGC-soma interactions. In the top figure of B, we found that the somas trigger a directional antagonism relationship with FGCs through *DDL3*, by which the somas inhibit FGCs whereas FGCs are neutral to the somas; Figure S4: (A) Fitting of power equation to the gene expression of four randomly chosen FGCs (a–d) against the expression indices of 1276 genes for early, middle, and late developmental stages of embryos 5 weeks, 14 weeks, and 23 weeks post-fertilization. (B) Plots of residuals from the fitting of the power equation vs. predicted expression values. The ticks at the x-axis indicate the expression indices of genes: Figure S5: Estimated expression profiles (slashed line) of a gene that mediates synergism (A), directional synergism (B), antagonism (C), directional antagonism (D), and altruism/exploitation (E), respectively, in a comparison to true profiles (solid line), under a sample size of 15 (left panel) and 50 (right panel). Red, green, and blue lines denote the overall, independent, and dependent expression profiles, respectively; Figure S6: Game and non-game models reciprocally analyze the expression data simulated by each model. Solid and slashed lines denote the true and estimated expression profiles, respectively. Red, green, and blue lines denote the overall, independent, and dependent expression profiles, respectively.

## References

[1] McLaughlin E.A., McIver S.C. (2009). Awakening the oocyte: Controlling primordial follicle development. Reproduction.

[2] Monniaux D., Clément F., Dalbiès-Tran R., Estienne A., Fabre S., Mansanet C., Monget P. (2014). The ovarian reserve of primordial follicles and the dynamic reserve of antral growing follicles: What is the link?. Biol. Reprod..

[3] Li L., Dong J., Yan L., Yong J., Liu X., Hu Y., Fan X., Wu X., Guo H., Wang X. (2017). Single-cell RNA-seq analysis maps development of human germline cells and gonadal niche interactions. Cell Stem Cell.

[4] Grive K.J., Freiman R.N. (2015). The developmental origins of the mammalian ovarian reserve. Development.

[5] Chen D., Sun N., Hou L., Kim R., Faith J., Aslanyan M., Tao Y., Zheng Y., Fu J., Liu W. (2019). Human primordial germ cells are specified from lineage-primed progenitors. Cell Rep..

[6] Canipari R. (2000). Oocyte--granulosa cell interactions. Hum. Reprod. Update.

[7] Cecconi S., Ciccarelli C., Barberi M., Macchiarelli G., Canipari R. (2004). Granulosa cell-oocyte interactions. Eur. J. Obstet. Gynecol. Reprod. Biol..

[8] Jemc J.C. (2011). Somatic gonadal cells: The supporting cast for the germline. Genesis.

[9] Saitou M., Miyauchi H. (2016). Gametogenesis from pluripotent stem cells. Cell Stem Cell.

[10] Cheng S., Pei Y., He L., Peng G., Reinius B., Tam P.P.L., Jing N., Deng Q. (2019). Single-cell RNA-seq reveals cellular heterogeneity of pluripotency transition and X chromosome dynamics during early mouse development. Cell Rep..

[11] Hedlund E., Deng Q. (2018). Single-cell RNA sequencing: Technical advancements and biological applications. Mol. Asp. Med..

[12] Shingleton A. (2010). Allometry: The study of biological scaling. Nat. Ed. Knowl..

[13] Brown J.H., Gillooly J.F., Allen A.P., Savage V.M., West G.B. (2004). Toward a metabolic theory of ecology. Ecology.

[14] von Neumann J., Morgenstern O. (1946). Theory of Games and Economic Behavior.

[15] Nash J.F. (1950). Equilibrium points in n-person games. Proc. Natl. Acad. Sci. USA.

[16] Smith J.M., Price G.R. (1973). The logic of animal conflict. Nature.

[17] Bomze I.M., Pötscher B.M. (1989). Game Theoretical Foundations of Evolutionary Stability.

[18] Cressman R., Tao Y. (2014). The replicator equation and other game dynamics. Proc. Natl. Acad. Sci. USA.

[19] Hart S., Mas-Colell A. (2003). Uncoupled dynamics do not lead to Nash equilibrium. Am. Econ. Rev..

[20] Hofbauer J., Sandholm W.H. (2009). Stable games and their dynamics. J. Econ. Theor..

[21] Fu L.Y., Sun L.D., Hao H., Jiang L.B., Zhu S., Ye M., Tang S., Huang M., Wu R.L. (2018). How trees allocate carbon for optimal growth: Insight from a game-theoretic model. Brief. Bioinform..

[22] Wang Q., Gosik K., Xing S., Jiang L.B., Sun L.D., Chinchilli V.M., Wu R.L. (2017). Epigenetic game theory: How to compute the epigenetic control of maternal-to-zygotic transition. Phys. Life Rev..

[23] Zomorrodi A.R., Segrè D. (2017). Genome-driven evolutionary game theory helps understand the rise of metabolic interdependencies in microbial communities. Nat. Commun..

[24] Guo F., Yan L., Guo H., Li L., Hu B., Zhao Y., Yong J., Hu Y., Wang X., Wei Y. (2015). The transcriptome and DNA methylome landscapes of human primordial germ cells. Cell.

[25] Chen C., Jiang L., Fu G., Wang M., Yang Y.Q., Shen B., Liu Z.Q., Wang Z.H., Hou W., Berceli S.A. (2019). An omnidirectional visualization model of personalized gene regulatory networks. NPJ Syst. Biol. Appl..

[26] Kumar M.P., Du J., Lagoudas G., Jiao Y., Sawyer A., Drummond D.C., Lauffenburger D.A., Raue A. (2018). Analysis of single-cell RNA-seq identifies cell-cell communication associated with tumor characteristics. Cell Rep..

[27] Kim B.-R., Zhang L., Berg A., Fan J., Wu R.L. (2008). A computational approach to the functional clustering of periodic gene expression profiles. Genetics.

[28] Callebaut W., Rasskin-Gutman D. (2009). Modularity: Understanding the Development and Evolution of Natural Complex Systems.

[29] Strogatz S.H. (2001). Exploring complex networks. Nature.

[30] Sinervo B., Lively C.M. (1996). The rock–paper–scissors game and the evolution of alternative male strategies. Nature.

[31] Shi Y., Massagué J. (2003). Mechanisms of TGF-beta signaling from cell membrane to the nucleus. Cell.

[32] James C., Ugo V., Le Couedic J.P., Staerk J., Delhommeau F., Lacout C., Garcon L., Raslova H., Berger R., Bennaceur-Griscelli A. (2005). A unique clonal JAK2 mutation leading to constitutive signalling causes polycythaemia vera. Nature.

[33] Bray S.J. (2006). Notch signalling: A simple pathway becomes complex. Nat. Rev. Mol. Cell Biol..

[34] Song X., Call G.B., Kirilly D., Xie T. (2007). Notch signaling controls germline stem cell niche formation in the Drosophila ovary. Development.

[35] Saitou M., Yamaji M. (2012). Primordial germ cells in mice. Cold Spring Harb. Perspect. Biol..

[36] Kurimoto K., Saitou M. (2015). Mechanism and reconstitution in vitro of germ cell development in mammals. Cold Spring Harbor Symp. Quant. Biol..

[37] Nowak M.A. (2006). Five rules for the evolution of cooperation. Science.

[38] Otte J., Wruck W., Adjaye J. (2017). New insights into human primordial germ cells and early embryonic development from single-cell analysis. FEBS Lett..

[39] Nguyen D.H., Jaszczak R.G., Laird D.J. (2019). Heterogeneity of primordial germ cells. Curr. Top. Dev. Biol..

[40] Griffin C., Jiang L., Wu R. (2020). Analysis of quasi-dynamic ordinary differential equations and the quasi-dynamic replicator. Phys. A Stat. Mech. Its Appl..

[41] Seah M.K.Y. (2018). Messerschmidt DM. From germline to soma: Epigenetic dynamics in the mouse preimplantation embryo. Curr. Top. Dev. Biol..

[42] Vo H., Wahiduzzaman, Tindell S.J., Zheng J.M., Gao M., Arkov A. (2019). Protein components of ribonucleoprotein granules from Drosophila germ cells oligomerize and show distinct spatial organization during germline development. Sci. Rep..

[43] Shannon P., Markiel A., Ozier O., Baliga N.S., Wang J.T., Ramage D., Amin N., Schwikowski B., Ideker T. (2003). Cytoscape: A software environment for integrated models of biomolecular interaction networks. Genome Res..

