# Supplementary material for: A Single-Cell Omics Network Model of Cell Crosstalk during the Formation of Primordial Follicles
Source: Cells. 2022 Jan 20;11(3):332. doi: 10.3390/cells11030332 (PMC8834074; doi:10.3390/cells11030332)
Supplement: Supplementary file 1 [file cells-11-00332-s001.zip › cells-1535038-supplementary.pdf]

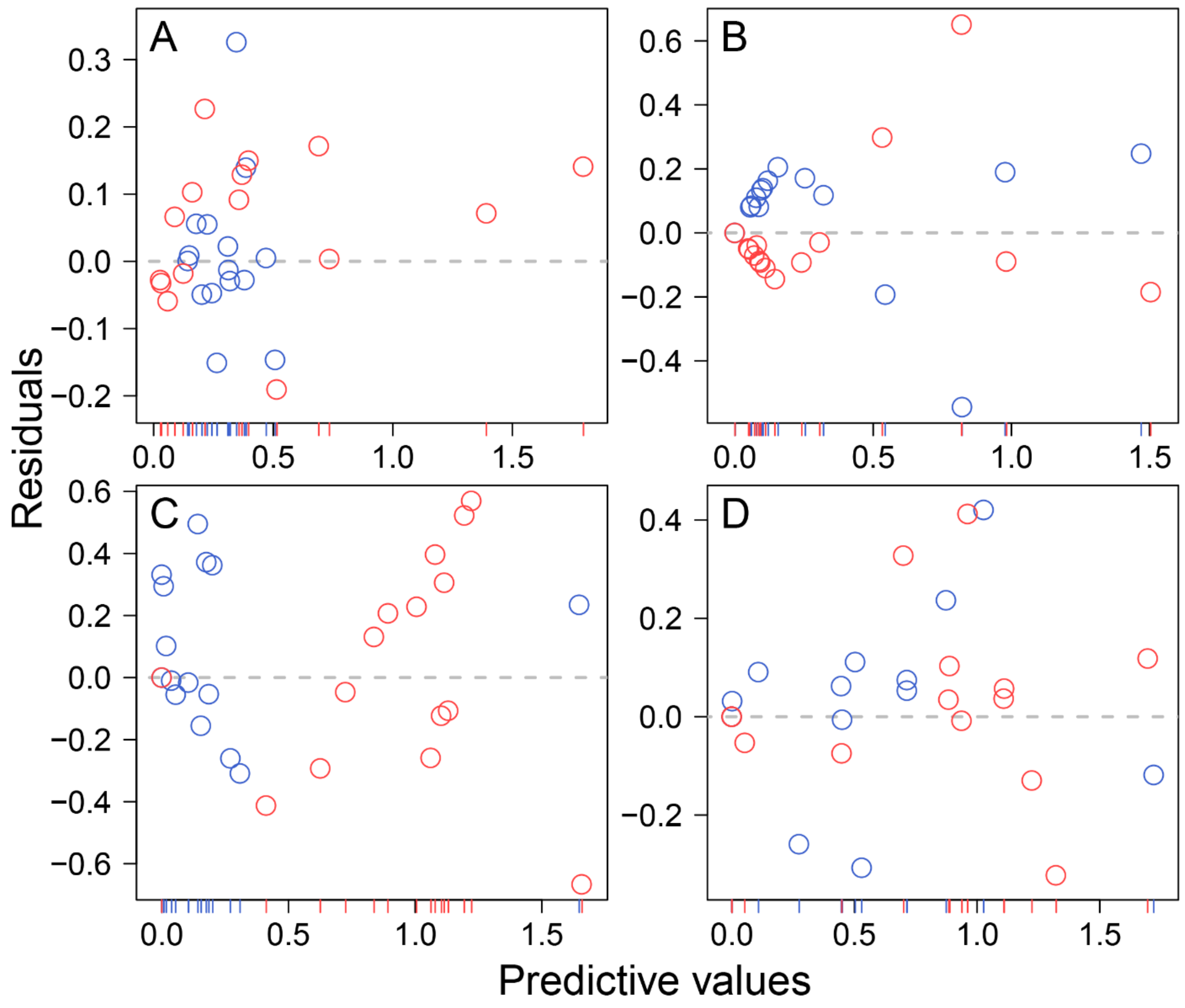

Figure S1 Plots of residuals from the fitting of the power equation vs. predicted expression values for four randomly chosen genes, *A2M* (**A**), *ADAP2* (**B**), *AKR1B10* (**C**), and *ANXA1* (**D**), for FGCs (red) and somas (blue). The ticks at the *x*-axis indicate the niche indices of embryos.

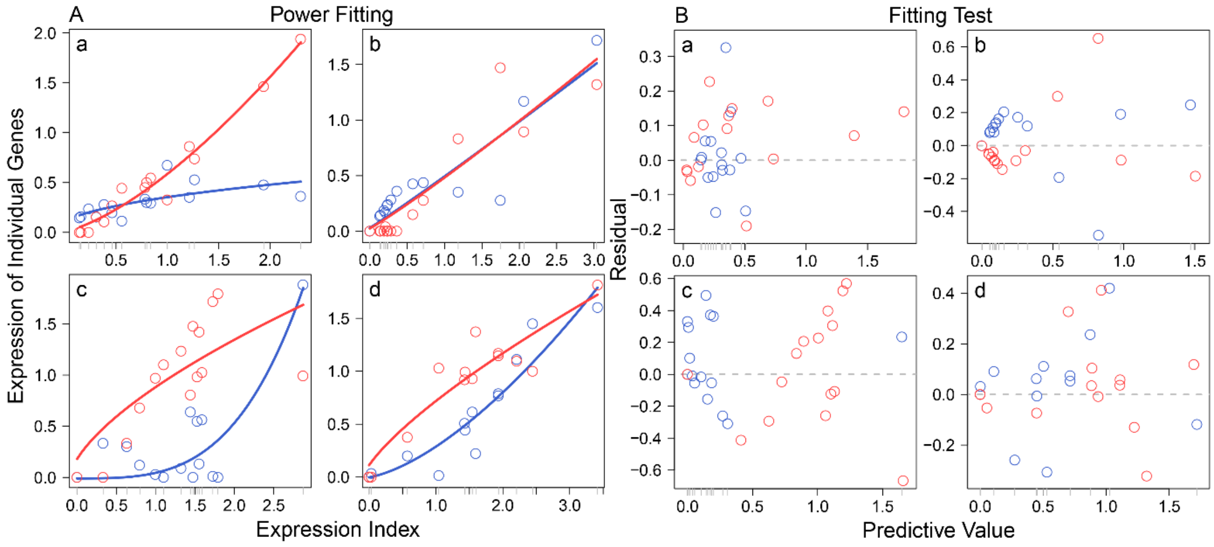

Figure S2. (**A**) Fitting of power equation to the expression of individual genes against expression indices for FGCs (blue) and somas (red) across embryos. (**B**) Plots of residuals from the fitting of the power equation vs. predicted expression values. As an example, we randomly chose genes, *A2M* (a), *ADAP2* (b), *AKR1B10* (c), and *ANXA* (d). The ticks at the *x*-axis indicate the expression indices of FGCs or somas.

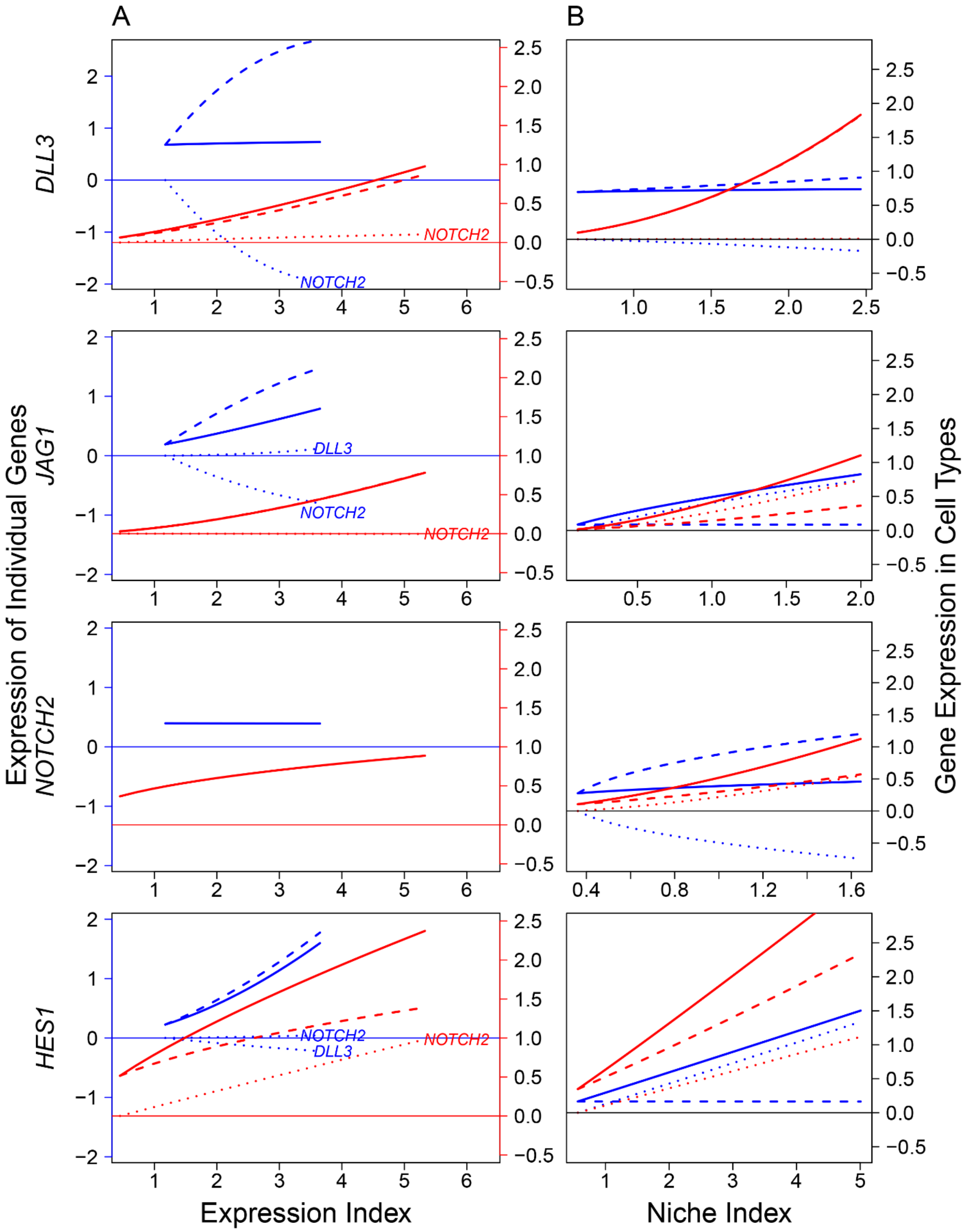

Figure S3 FGC-soma interactions driven by genes involved in the NOTCH signaling pathway, where *DLL3* is the ligand highly expressed in all phases of FGCs and *JAG1* is the ligand specifically expressed in oogenesis phase FGCs, *NOTCH2* is the receptor and *HES1* is the target,

both of which are highly expressed in nearly all FGCs. (**A**) *DLL3*, *JAG1*, *NOTCH2*, and *HES1* work together in a network to mediate FGC and soma development as a function of expression index, respectively. Left and right sides of the figure denote the expression levels of genes in FGCs (blue) and the somas (red), respectively. (**B**) The niche index-varying pattern of interactions between FGCs (blue) and somas (red) driven by individual genes. Solid thick lines, slash lines, and dots lines denote the overall expression level, independent expression level, and dependent expression level, respectively. Ligand *DLL3* is expressed both in FGCs and somatic cells, suggesting that it provides a foundation for NOTCH signaling interaction between these two different types of cells. Our qdODE model can dissect how DDL3 determine FGC-soma interactions. In the top figure of B, we found that the somas trigger a directional antagonism relationship with FGCs through DDL3, by which the somas inhibit FGCs whereas FGCs are neutral to the somas.

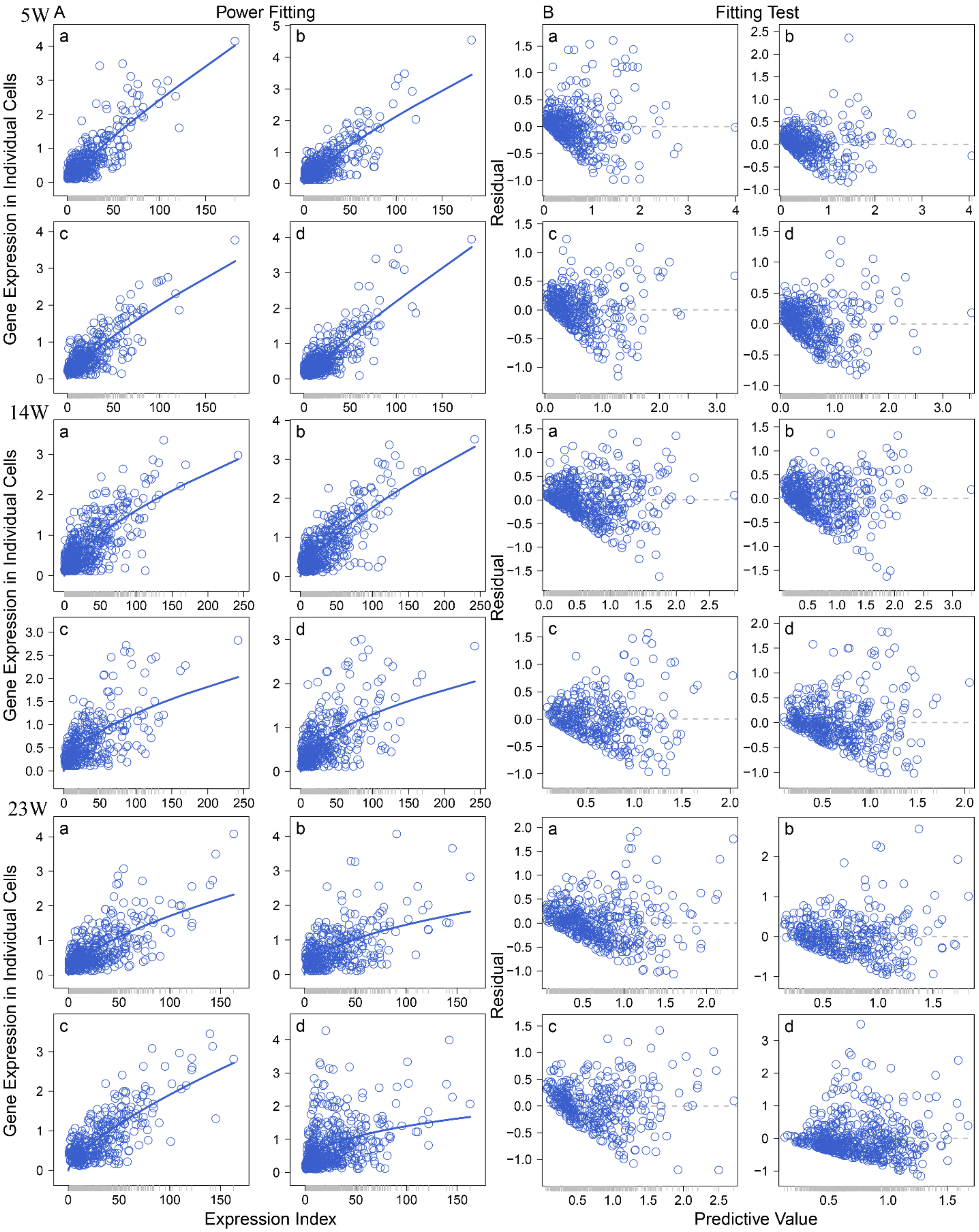

Figure S4. (**A**) Fitting of power equation to the gene expression of four randomly chosen FGCs (a – d) against the expression indices of 1,276 genes for early, middle, and late developmental

stages of embryos 5 w, 14 w, and 23 w post-fertilization. (**B**) Plots of residuals from the fitting of the power equation vs. predicted expression values. The ticks at the *x*-axis indicate the expression indices of genes.

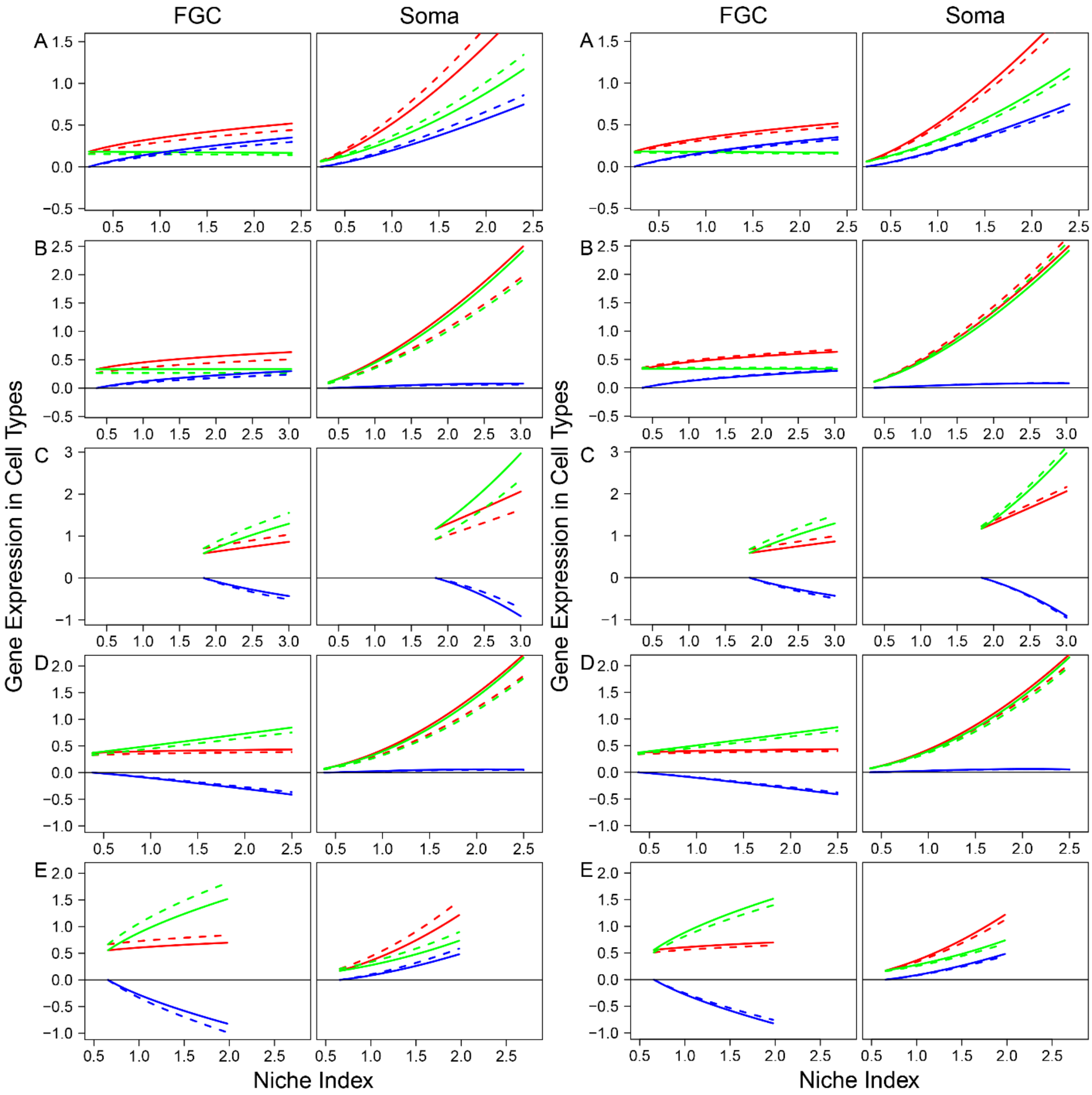

Figure S5 Estimated expression profiles (slashed line) of a gene that mediates synergism (**A**), directional synergism (**B**), antagonism (**C**), directional antagonism (**D**), and altruism/exploitation (**E**), respectively, in a comparison to true profiles (solid line), under a sample size of 15 (left panel) and 50 (right panel). Red, green, and blue lines denote the overall, independent, and dependent expression profiles, respectively.

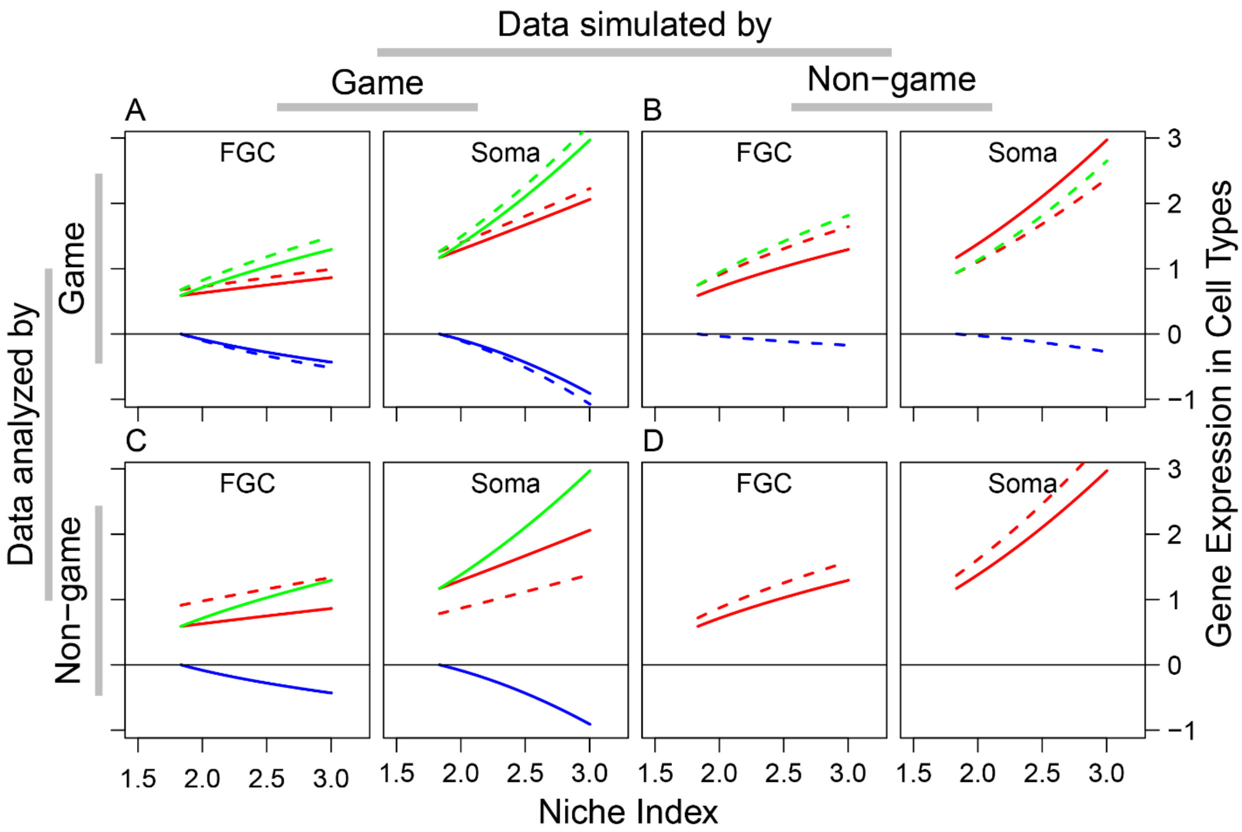

Figure S6 Game and non-game models reciprocally analyze the expression data simulated by each model. Solid and slashed lines denote the true and estimated expression profiles, respectively. Red, green, and blue lines denote the overall, independent, and dependent expression profiles, respectively.
